# Modeling the Growth and Interaction Between *Brochothrix thermosphacta*, *Pseudomonas* spp., and *Leuconostoc gelidum* in Minced Pork Samples

**DOI:** 10.3389/fmicb.2020.00639

**Published:** 2020-04-09

**Authors:** Emilie Cauchie, Laurent Delhalle, Ghislain Baré, Assia Tahiri, Bernard Taminiau, Nicolas Korsak, Sophie Burteau, Papa Abdoulaye Fall, Frédéric Farnir, Georges Daube

**Affiliations:** ^1^Department of Food Sciences, Fundamental and Applied Research for Animal and Health, Faculty of Veterinary Medicine, University of Liège, Liège, Belgium; ^2^Quality Partner sa, Liège, Belgium

**Keywords:** predictive microbiology, growth parameters, interaction models, *Brochothrix thermosphacta*, *Pseudomonas* spp., *Leuconostoc gelidum*, Jameson-effect model, Lotka Volterra model

## Abstract

The aim of this study was to obtain the growth parameters of specific spoilage micro-organisms previously isolated in minced pork (MP) samples and to develop a three-spoilage species interaction model under different storage conditions. Naturally contaminated samples were used to validate this approach by considering the effect of the food microbiota. Three groups of bacteria were inoculated on irradiated samples, in mono- and in co-culture experiments (*n* = 1152): *Brochothrix thermosphacta*, *Leuconostoc gelidum*, and *Pseudomonas* spp. (*Pseudomonas fluorescens* and *Pseudomonas fragi*). Samples were stored in two food packaging [food wrap and modified atmosphere packaging (CO_2_ 30%/O_2_ 70%)] at three isothermal conditions (4, 8, and 12°C). Analysis was carried out by using both 16S rRNA gene amplicon sequencing and classical microbiology in order to estimate bacterial counts during the storage period. Growth parameters were obtained by fitting primary (Baranyi) and secondary (square root) models. The food packaging shows the highest impact on bacterial growth rates, which in turn have the strongest influence on the shelf life of food products. Based on these results, a three-spoilage species interaction model was developed by using the modified Jameson-effect model and the Lotka Volterra (prey–predator) model. The modified Jameson-effect model showed slightly better performances, with 40–86% out of the observed counts falling into the Acceptable Simulation Zone (ASZ). It only concerns 14–48% for the prey–predator approach. These results can be explained by the fact that the dynamics of experimental and validation datasets seems to follow a Jameson behavior. On the other hand, the Lotka Volterra model is based on complex interaction factors, which are included in highly variable intervals. More datasets are probably needed to obtained reliable factors, and so better model fittings, especially for three- or more-spoilage species interaction models. Further studies are also needed to better understand the interaction of spoilage bacteria between them and in the presence of natural microbiota.

## Introduction

During production and distribution steps, spoilage of meat and meat products may occur, rendering them unacceptable for human food consumption. Spoilage is mainly caused by microbial growth, which triggers alterations in the sensorial qualities of the product, with off-odor and off-flavor, discoloration, texture changes, etc. (Kreyenschmidt et al., 2010; Dalcanton et al., 2013; Pinter et al., 2014; Cauchie et al., 2017; Den Besten et al., 2017; Torngren et al., 2018). It is well known that the initial bacterial counts on meat and meat products is highly variable (Benson et al., 2014), but several studies have established that only a dominant fraction of the microbiota, designated as specific spoilage organisms (SSOs), contributes to spoilage (Nychas et al., 2008; Kreyenschmidt et al., 2010; Pennacchia et al., 2011; Benson et al., 2014; Zotta et al., 2019). In this context, predictive microbiology can be a helpful tool because the prediction of microbial growth, especially SSOs, enables food industries to optimize their production and storage managements, and thus reduce their economic losses (Kreyenschmidt et al., 2010; Fakruddin et al., 2012; Li et al., 2017; Tamplin, 2018).

During the last years, several models have been developed to predict the growth of SSOs in meat and meat products (Liu et al., 2006; Mataragas et al., 2006; Koutsoumanis, 2009; Kreyenschmidt et al., 2010; Dalcanton et al., 2013; Mejlholm and Dalgaard, 2013). But the majority of the developed models are based on the growth of two bacterial species in a food matrix (Vereecken et al., 2000; Giuffrida et al., 2007), most often to study the interaction between spoilage and pathogenic bacteria (Lebert et al., 2000; Mejlholm and Dalgaard, 2007; Giuffrida et al., 2009; Cornu et al., 2011; Ye et al., 2014; Correia Peres Costa et al., 2019; Pedrozo et al., 2019). Moreover, these models often describe the growth of the SSOs depending on the storage temperature (Dominguez and Schaffner, 2007; Gospavic et al., 2008; Kreyenschmidt et al., 2010; Psomas et al., 2011; Longhi et al., 2013; Antunes-Rohling et al., 2019) or the packaging conditions (Devlieghere et al., 1999; Chaix et al., 2015; Guillard et al., 2016; Couvert et al., 2019; Kapetanakou et al., 2019), but do not always consider the interaction of these storage conditions for the growth of spoilage bacteria (Rosso et al., 1995; Augustin and Carlier, 2000; Le Marc et al., 2002; Pinon et al., 2004; Dalcanton et al., 2018; Kakagianni et al., 2018; Nyhan et al., 2018; Correia Peres Costa et al., 2019).

As mentioned by Correia Peres Costa et al. (2019): “interaction models are usually intended to quantify how much the growth of one population is reduced by the growth of other populations.” In this context, two model approaches are generally used to describe the microbial interaction: (i) those based on the modified Jameson-effect phenomenon (Jameson, 1962; Cornu et al., 2011; Ye et al., 2014; Cauchie et al., 2017; Correia Peres Costa et al., 2019), and (ii) those based on the predator-prey models (Lotka Volterra equation) (Dens et al., 1999; Berlow et al., 2004; Powell et al., 2004; Giuffrida et al., 2007; Mounier et al., 2008; Cornu et al., 2011; Ye et al., 2014; Correia Peres Costa et al., 2019).

As described by Cornu et al. (2011), the Jameson-effect model assumes that: “(i) many microbial interactions in foods limit the maximum population density, without any significant effect on the lag time, and (ii) the growth of the minority population is only partly inhibited after the majority population count has reached its stationary phase [maximum critical population, *MCP*, expressed in log colony forming units (CFU)/g].” The modified Jameson-effect model makes the hypothesis that there is one single inhibition function for both populations; hence, both populations are similarly inhibited by the same limiting resource, the same waste products, and/or by change in pH (Cornu et al., 2011). Recently, Quinto et al. (2018) have developed a three-strain model based on the modified Jameson-effect equation for inoculated spoilage and pathogenic bacteria in a reconstituted sterile skimmed milk. This study considers the effect of two bacteria, *Pseudomonas fluorescens* and *Listeria innocua*, on the bacterial growth of *Listeria monocytogenes*. But the effect of the natural food microbiota on the growth of specific spoilage bacteria needs to be studied (Rouger et al., 2017) in order to predict bacterial growth resulting from several interactions between three or more spoilage species (Ye et al., 2014). This approach needs to be studied.

The Lotka Volterra model can be considered as a prey-predator model that includes competition for a common substrate (Cornu et al., 2011). As cited by Chauvet et al. (2002), the Lotka Volterra model for a three-species food chain approach can be considered as: “the lowest-level prey *x* is preyed upon by a mid-level species *y*, which, in turn, is preyed upon by a top-level predator *z.*” However, this hypothesis cannot always be applied in food matrix. Indeed, the growth of a bacterium (*B*_*A*_) presents simultaneously with other bacteria in a food matrix (*B*_*B*_ and *B*_*C*_) can be affected by three different ways: (i) *B*_*A*_ growth with a reduced growth rate after that *B*_*B*_ and *B*_*C*_ reach their maximal population densities (*N*_*max*_, expressed in log CFU/g), (ii) *B*_*A*_ stops growing when *B*_*B*_ and *B*_*C*_ reach their *N*_*max*_, and (iii) *B*_*A*_ declines when *B*_*B*_ and *B*_*C*_ reach their *N*_*max*_ (Cauchie et al., 2017; Correia Peres Costa et al., 2019). It could be so interesting to develop a Lotka Volterra model for a three-species approach, by considering the effect of the natural food microbiota for the growth of specific spoilage bacteria. Also, this approach is, to the best knowledge of the authors, not available in the literature.

Based on these, the objectives of the present study were (i) to obtain the growth parameters of three specific spoilage micro-organisms previously isolated in minced pork (MP) samples, according to different storage conditions, (ii) to develop a three-spoilage species interaction model based on available models, under food wrap and modified atmosphere packaging, at isothermal conditions, and (iii) to validate this approach with naturally contaminated food samples stored under different storage conditions.

## Materials and Methods

### Sampling

Fresh MP samples were obtained from a local Belgian manufacturer at the day of the production, corresponding to the day of slaughtering. MP samples were packed by the manufacturer in a polypropylene tray under cling film (high film permeability).

According to the recipe, MP is composed of 100% pork mince (70% lean, 30% fat), no salt, no spices, no additives, no eggs, and no sugar are added.

At the day of the production, the water activity of the product was 0.98 ± 0.02 and the pH value was 5.80 ± 0.05 (*n* = 12). pH of the homogenized samples (5 g in 45 mL of KCl) was measured with a pH meter (Knick 765 Calimatic, Allemagne). The water activity was measured for homogenized samples on the basis of the relative humidity measurement of the air balance in the micro enclosure at 25 ± 0.4°C (Thermoconstanter TH200, Novasina, Switzerland).

Food samples were then stored at −20°C and irradiated by gamma irradiation (17.5 ± 0.4 kGy) at the same temperature (Sterigenics, Fleurus, Belgium) to limit the adverse effects of irradiation at this dose (Kim et al., 2002; Ham et al., 2017; Wang et al., 2018).

### Bacterial Strains

As described in the study of Cauchie et al. (2019), three specific spoilage micro-organisms were previously isolated from different batches of naturally contaminated Belgian MP samples at the end of their use-by date. Samples were stored under two packaging (under air and modified atmosphere—30% CO_2_–70% O_2_) and three temperature conditions (4, 8, and 12°C). These predominant strains, represented more than 50% of the natural microbiota, were identified by 16S rRNA sequencing and used for experiments: *Brochothrix thermosphacta* (MM008), *Leuconostoc gelidum* (MM045) *Pseudomonas* spp. (*P. fluorescens* MM026 and *Pseudomonas fragi* MM014). *P. fluorescens* and *P. fragi* were used together because experiments were carried out in an exploratory approach to the proposed method, thus wishing to consider a wide diversity of *Pseudomonas* species most frequently found in MP.

*Brochothrix thermosphacta* MM008, *L. gelidum* (MM045), *P. fragi* MM014, and *P. fluorescens* MM026 were stored at −80°C in nutrient broth with 30% glycerol as a cryoprotective agent. Before use, strains were transferred from the −80°C culture collection to Brain Heart Infusion (BHI) broth for 48 h at 22°C. The bacterial suspensions were incubated overnight at 4°C before inoculation at stationary phase (7.00 log CFU/mL).

### Inoculation Experiments

The three selected bacteria suspensions were inoculated on irradiated MP samples (1% v/w), in triplicate, for mono-culture and co-culture experiments with the objective to reach an average concentration of 3.0 log CFU/g (on the product).

Mono-culture experiments were performed by inoculation of individual bacterial strains: *B. thermosphacta* MM008, *Pseudomonas* spp. (*P. fluorescens* MM026, *P. fragi* MM014, 1:1 ratio), and *L. gelidum* MM045.

Co-culture experiments were performed by inoculation of a mix containing *B. thermosphacta* MM008, Pseudomonas spp. (*P. fragi* MM0014 and *P. fluorescens* MM0026, 1:1 ratio), and *L. gelidum* MM045 (1:1:1 ratio).

Non-inoculated control samples were homogenized, in triplicate, by adding the same quantity of sterile water only.

After inoculation, MP samples were mixed in a Kenwood mixer for 2 min in speed 2 (Kenwood, Mechelen, Belgium).

Inoculated and non-inoculated MP samples were then packed (50 g) in two different types of non-sterile packaging. The first packaging was a high barrier tray (187 × 137 × 36, polyester 10 μm, homo-polymer polypropylene 50 μm, NutriPack, France) under modified atmosphere (MAP, CO_2_ 30%/O_2_ 70% ± 0.1%) (Olympia V/G, Technovac, Italy) using packaging wrap (PP/EVOH/PP) with random gas measurements (CheckMate 3, Dansensor, France). The second packaging concerns a weak barrier tray (175 × 135 × 22, polystyrene) under food wrap packing (FW) using cling film (Clinofilm).

In this study, MP samples were stored during a 13-days shelf life at isothermal temperature: (i) 4°C (±1°C), (ii) 8°C (±1°C), (iii) and 12°C (±1°C), in climatic chambers (Sanyo MIR 254) (288 samples for four experiments, *n* = 1152 samples) (Supplementary Figure S1). A storage time of 13 days was defined in this study in order to obtain a sufficient number of points for modeling, allowing us to predict all the growth phases.

The codes used for each experiment, depending on the inoculated bacteria and storage conditions, are listed in Table 1.

**TABLE 1:** List of the codes used for the experiments, depending on the inoculated bacteria and storage conditions.

Experiments	Food packaging	Temperature (°C)	Bacterial species	Codes
Mono-culture	FW	4	*B. thermosphacta*	*A*_*mono*_
	FW	8		*B*_*mono*_
	FW	12		*C*_*mono*_
	MAP	4		*D*_*mono*_
	MAP	8		*E*_*mono*_
	MAP	12		*F*_*mono*_
Mono-culture	FW	4	*Pseudomonas* spp.	*G*_*mono*_
	FW	8		*H*_*mono*_
	FW	12		*I*_*mono*_
	MAP	4		*J*_*mono*_
	MAP	8		*K*_*mono*_
	MAP	12		*L*_*mono*_
Mono-culture	FW	4	*L. gelidum*	*M*_*mono*_
	FW	8		*N*_*mono*_
	FW	12		*O*_*mono*_
	MAP	4		*P*_*mono*_
	MAP	8		*Q*_*mono*_
	MAP	12		*R*_*mono*_
Co-culture	FW	4	*B. thermosphacta*	*A*_*co(A)*_
			*Pseudomonas* spp.	*A*_*co(B)*_
			*L. gelidum*	*A*_*co(C)*_
	FW	8	*B. thermosphacta*	*B*_*co(A)*_
			*Pseudomonas* spp.	*B*_*co(B)*_
			*L. gelidum*	*B*_*co(C)*_
	FW	12	*B. thermosphacta*	*C*_*co(A)*_
			*Pseudomonas* spp.	*C*_*co(B)*_
			*L. gelidum*	*C*_*co(C)*_
	MAP	4	*B. thermosphacta*	*D*_*co(A)*_
			*Pseudomonas* spp.	*D*_*co(B)*_
			*L. gelidum*	*D*_*co(C)*_
	MAP	8	*B. thermosphacta*	*E*_*co(A)*_
			*Pseudomonas* spp.	*E*_*co(B)*_
			*L. gelidum*	*E*_*co(C)*_
	MAP	12	*B. thermosphacta*	*F*_*co(A)*_
			*Pseudomonas* spp.	*F*_*co(B)*_
			*L. gelidum*	*F*_*co(C)*_

*FW, food wrap; MAP, modified atmosphere packaging (CO_2_ 30%/O_2_ 70% ± 0.1%); mono, mono-culture experiments; co, co-culture experiments with by individually tracking the inoculated bacteria by metagenetic analysis [B. thermosphacta, _co__(A)_; Pseudomonas spp., _co(B)_; L. gelidum, _co(C)_].*

### pH and Gas Composition Measurements

At the first and the last day of storage, pH of the homogenized samples (5 g in 45 mL of KCl) was measured with a pH meter (Knick 765 Calimatic, Allemagne).

Oxygen and carbon oxygen concentrations of samples stored in modified atmosphere packaging were monitored daily (CheckMate 3, Dansesor, France).

Non-parametric statistical tests were used to compare the pH values and the gas measurements between samples. All tests were considered as significant for a *p*-value < 0.05.

### Plate Count Enumeration

Twenty-five grams of product were put into a Stomacher bag with a mesh screen liner (80 μm pore size) (Biomérieux, Basingstoke, England, ref 80015) under aseptic conditions. Buffered peptone water (BPW, 10 g/L peptone, 5 g/L sodium chloride, #3564684, Bio-Rad, Marnes-la-Coquette, France) (225 mL) was automatically added to each bag (Dilumat, Biomérieux, Belgium) and the samples were homogenized for 2 min in a Stomacher (Bagmixer, Interscience, France). From this primary suspension, decimal dilutions in maximum recovery diluent (1.0 g/L peptone, 8.5 g/L sodium chloride, #CM0733, Oxoid, Hampshire, England) were prepared for microbiological analysis, and 0.1 mL aliquots of the appropriate dilutions were plated onto media for each analysis (Spiral plater, DW Scientific, England).

Total viable counts (TVCs) for the aerobic psychrophilic microbiota were enumerated on plate count agar (PCA agar, #3544475, Bio-Rad, Marnes-la-Coquette, France) after 72 h at 22°C (model 1535 incubator, Shel Lab, Sheldon Manufacturing, Inc., United States).

Plate counts were performed for mono- and co-culture experiments, and transformed in decimal logarithmic values. Samples for both experiments were enumerated at the first day of inoculation (day 0) and daily until the last day of storage (day 13). None specific agar media were used in co-culture experiments to separately enumerate the three inoculated species. Non-inoculated control samples were analyzed at day 0 and at day 13.

Using R software (R Core Team, 2019), an analysis of covariance (ANCOVA) was performed to evaluate the effect of the storage conditions on plate counts (FactoMineR package, Le et al., 2008). All tests were considered as significant for a *p*-value < 0.05.

### 16S rDNA Metagenetic Approach

A 16S rDNA metagenetic approach was used for mono- and co-culture experiments.

In mono-culture experiments, metagenetic analysis were performed at the first day of inoculation (day 0) and at the last day of storage (day 13) for samples stored at 4°C.

In co-culture experiments, samples were analyzed at day 0 and daily until day 13. The results were then correlated with plate counts in order to obtain estimate bacterial abundance over storage (see section “16S rDNA Data Analysis and Bacterial Abundance”).

No 16S rDNA metagenetic analysis was performed for non-inoculated control samples.

#### DNA Extraction and 16S rDNA Amplicon Sequencing

Bacterial DNA was extracted from each primary suspension, previously stored at –80°C, using the DNEasy Blood and Tissue kit (QIAGEN Benelux BV, Antwerp, Belgium) following the manufacturer’s recommendations. The resulting DNA extracts were eluted in DNAse/RNAse free water and their concentration and purity were evaluated by means of optical density using the NanoDrop ND-1000 spectrophotometer (Isogen, St-Pieters-Leeuw, Belgium). DNA samples were stored at –20°C until used for 16S rDNA amplicon sequencing.

PCR-amplification of the V1–V3 region of the 16S rDNA library preparation was performed with the following primers (with Illumina overhand adapters), forward (5′-GAGAGTTTGA TYMTGGCTCAG-3′) and reverse (5′-ACCGCGGCTGCTGG CAC-3′). Each PCR product was purified with the Agencourt AMPure XP beads kit (Beckman Coulter; Pasadena, CA, United States) and submitted to a second PCR round for indexing, using the Nextera XT index primers 1 and 2. Thermocycling conditions consisted of a denaturation step of 4 min at 94°C, followed by 25 cycles of denaturation (15 s at 94°C), annealing (45 s at 56°C), and extension (60 s at 72°C), with a final elongation step (8 min at 72°C). These amplifications were performed on an EP Mastercycler Gradient System device (Eppendorf, Hamburg, Germany). The PCR products of approximately 650 nucleotides were run on 1% agarose gel electrophoresis and the DNA fragments were plugged out and purified using a Wizard SV PCR purification kit (Promega Benelux, Leiden, Netherlands). After purification, PCR products were quantified using the Quanti-IT PicoGreen (ThermoFisher Scientific, Waltham, MA, United States) and diluted to 10 ng/μL. A final quantification, by quantitative (q)PCR, of each sample in the library was performed using the KAPA SYBR^®^ FAST quantitative PCR (qPCR) Kit (KapaBiosystems, Wilmington, MA, United States) before normalization, pooling, and sequencing on a MiSeq sequencer using V3 reagents (Illumina, San Diego, CA, United States).

#### Bioinformatics Analysis

The 16S rRNA gene sequence reads were processed with MOTHUR. The quality of all sequence reads was denoised using the Pyronoise algorithm implemented in MOTHUR. The sequences were checked for the presence of chimeric amplification using ChimeraSlayer (developed by the Broad Institute^1^). The obtained read sets were compared to a reference dataset of aligned sequences of the corresponding region derived from the SILVA database of full-length rRNA gene sequences^2^ (version v1.2.11) implemented in MOTHUR. The final reads were clustered into operational taxonomic units (OTUs), using the nearest neighbor algorithm using MOTHUR with a 0.03 distance unit cutoff. A taxonomic identity was attributed to each OTU by comparison to the SILVA database, using an 80% homogeneity cutoff. As MOTHUR is not dedicated to the taxonomic assignment beyond the genus level, all unique sequences for each OTU were compared to the SILVA dataset 111, using a BLASTN algorithm. For each OTU, a consensus detailed taxonomic identification was given based upon the identity (<1% mismatch with the aligned sequence) and the metadata associated with the best hit (validated bacterial species or not).

#### 16S rDNA Data Analysis and Bacterial Abundance

A correcting factor for 16S rDNA gene copy numbers was applied for any taxon *i* (Eq. 1).

(1) A=iNk/Ci

Where *A*_*i*_ is the real abundance of 16S genes from the taxon in the sample, *N*_*k*_ is the number of reads for the taxon in the sample *k*, and *C*_*i*_ is determined by the genomic 16S copy number of that taxon. To obtain each gene copy number, Ribosomal RNA Database (rrnDB) (Stoddard et al., 2015) and EzBioCloud database (Yoon et al., 2017) were used.

Then, to compare the relative abundance of OTUs, the number of reads of each taxon was normalized as described by Chaillou et al. (2015). Reads counts of each taxon *i* in the sample *k* were divided by a sample-specific scaling factor (*Si*) (Eq. 2) (Fougy et al., 2016; Rouger et al., 2018):

(2) $${\mathrm{Nr}}_{i}=A_{i}/S_{k}$$

Where *Nr*_*i*_ is the normalized number of reads for the taxon in the sample, *A*_*i*_ is the real abundance of 16S rRNA genes from that taxon obtained with a correcting factor for 16S rRNA gene copy numbers, and *S*_*k*_ is the normalization factor associated with sample *k*.

The sample-specific scaling factor was calculated by (Eq. 3):

(3) $$S_{k}=T_{k}/m_{e}$$

Where *S*_*k*_ is the sample-specific scaling factor associated with sample *k*, *T*_*k*_ is the number of total reads in the sample *k*, and *m*_*e*_ is the median value of total reads for all the samples of the dataset. Reads counts of all samples were then transformed into a percentage of each OTU.

For co-culture experiments, the percentage of each OTUs was finally converted as a proportion of the TVC, obtained by classical microbiological analysis, in order to estimate counts for each species [in log_10_ CFU/g, and expressed as mean ± standard deviation (SD)] (Eq. 4), as described by Cauchie et al. (2017).

(4) $$C_{\mathrm{bacterial species}}=(C_{\mathrm{total microbiota}}\times P_{\mathrm{reads of bacterial species}})/\mathrm{100}\;(4)$$

Where *C*_*bacterial species*_ is the estimated abundance concentration in the sample (log CFU/g), *C*_*total microbiota*_ is the bacterial concentration per samples in the PCA analysis (log CFU/g), and *P*_*reads of bacterial species*_ is the proportion of reads for the bacterial species per sample in the metagenetic analysis (expressed in% of the total number reads in the sample).

All biosample raw reads were deposited at the National Center for Biotechnology Information (NCBI) and are available under de BioProject ID PRJNA590608. The raw data supporting the conclusions of this article will be made available by EC to any qualified researcher.

### Approach Used to Develop the Interaction Model

**FIGURE 1:**
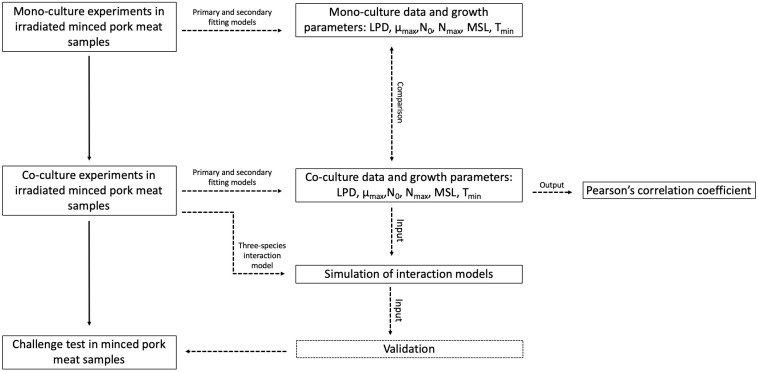
Schematic overview of the step-wise method used for the development of a three-spoilage species interaction model.

As proposed by Correia Peres Costa et al. (2019), a step-wise approach (Figure 1) was followed to develop interaction models simulating the growth of specific spoilage micro-organisms.

First, primary and secondary models were performed on mono-culture experiments to obtain the kinetic parameters (section “Primary and Secondary Model for the Fitting of Experimental Data”): lag phase duration (*LPD*, hours), maximum specific growth rate (μ_*max*_, 1/hours), initial and maximal population densities (*N*_0_ and *N*_*max*_, respectively, log CFU/g), theoretical minimal temperature of growth (*T*_*min*_,°C), growth rate obtained at the reference temperature of 20°C (μ_*ref*_, 1/hours), and minimal shelf life (*MSL*). The *MSL* is the time for the plate counts reaching approximatively 7.0 log CFU/g (expressed as Spoilage value according to the scientific literature, *S*_*val*_).

Second, the same approach was applied for co-culture experiments in order to obtain the growth parameters (section “Primary and Secondary Model for the Fitting of Experimental Data”), and to compare them with those on mono-culture experiments (section “Correlations Between Growth Parameters”). The Pearson’s correlation coefficient was also used to choose the highest influencing growth parameters on the microbial shelf life of MP samples (section “Correlations Between Growth Parameters”).

Third, all of these results were used to estimate competitions parameters in interaction models for a three-species approach, based on the modified Jameson-effect model and Lotka Volterra model (section “Modeling Microbial Interactions for *B. thermosphacta*, *Pseudomonas* spp., and *L. gelidum*”).

Finally, validation of growth and interaction parameters obtained by the three-species models was performed with naturally contaminated MP samples stored under different conditions (section “Model Validation”).

#### Primary and Secondary Model for the Fitting of Experimental Data

The primary model of Baranyi and Roberts (1994) (Eq. 5) was fitted to the experiment dataset obtained for mono- and co-culture experiments. Experimental dataset is obtained by plate counts in mono-culture, and by estimate abundance based on metagenetic results in co-culture. All the data from the three replicates were modeled.

Based on primary fitting, the growth kinetic parameters were obtained.

(5) $$N_{t}=N_{0}+\mu_{\max}\times A_{t}+\ln[1+\frac{\exp(\mu_{\max}\times A_{t)}-1}{\exp(N_{\max}-N_{0})}]$$

Where *N*_*t*_ the bacterial population at any time *t* (log CFU/g); *N*_*max*_ and *N*_0_, the maximum and initial population level, respectively (log CFU/g); μ_*max*_, the maximum specific growth rate (1/hour); and *A*_*t*_, an adjustment function to define the *LPD* (Eq. 6).

(6) $$\begin{array}{c} A_{t}=t+\frac{1}{\mu_{\max}}\times \ln\{\exp\left(-\mu_{\max}\times t\right)\\ \;+\exp\left(-h_{0}\right)-\exp\left[\left(-\mu_{\max}\times t\right)-h_{0}\right]\} \end{array}$$

Where *h*_0_ is simply a transformation of the initial conditions.

All fittings were performed using the nlsMicrobio package (function: baranyi, Baty and Delignette-Muller, 2013) from the open source R software (R Core Team, 2019).

The adequacy of the primary models to describe the experimental data was observed by using the root-mean-square error of the residuals (*RrMSE*, SD of the residuals) (Eq. 7) and the coefficient of multiple determination (*R*^2^, the fraction of the square of the deviations of the observed values about their mean explained by the equation fitted to the experimental data) (Eq. 8).

(7) $$RrMSE=\sqrt{\frac{RSS}{DF}}=\frac{\sum_{i=1}^{n}{\left(x_{i}^{0}-x_{i}^{f}\right)}^{2}}{n-s}$$

Where *RSS*, the residual sum of square; *DF*, the degrees of freedom; *n*, the number of data points; *s*, the number of parameters of the model; *x*_*i*_^0^, the observed values; and *x*_*i*_^*f*^, the fitted values.

(8) $$R^{2}=1-\frac{\sum_{i=1}^{n}{\left(observed_{i}-predicted_{i}\right)}^{2}}{\sum_{i=1}^{n}{\left(observed_{i}-mean\right)}^{2}}$$

Where *n*, the total number of data points; *mean*, the average value from all observed values.

A reparameterized version of the square root secondary model (Ratkowsky et al., 1982) (Eq. 9) was then used in R (R Core Team, 2019) to assess the effects of temperature on the growth rates.

(9) $$\mu_{\text{max}}=\mu_{\text{ref}}{\left(\frac{\text{T}-{\text{T}}_{\text{min}}}{{\text{T}}_{\text{ref}}-{\text{T}}_{\text{min}}}\right)}^{2}$$

Where μ_*ref*_ is the reference growth rate obtained at *T*_*ref*_ = 20°C (1/hours), *T* is the temperature (°C), and *T*_*min*_ is the minimal temperature for growth (°C) found in the scientific literature for the studied bacterial species: −3.36°C for *B. thermosphacta* (Leroi et al., 2012); −5.00°C for *Pseudomonas* spp. (Rashid et al., 2001); and + 1.00°C for *L. gelidum* (Kim et al., 2000).

For comparison, *T*_*min*_ values were also estimated by the Rosso primary model (Rosso et al., 1995) and the square root model (Ratkowsky et al., 1983) (Eq. 10).

(10) $$\sqrt{\mu_{max}}=btimes\left(T-T_{min}\right)$$

Where μ_*max*_ is the maximal growth rate (1/hours), *b* is a constant parameter obtained by linear regression, *T* is the temperature (°C), and *T*_*min*_ is the minimal temperature for growth (°C).

For secondary models, the coefficient of multiple determination (*R*^2^) and the goodness of fit (*GoF*, root-meat-square error of the model, analogous to the accuracy factor) were used (Eq. 11).

(11) $$GoF=\frac{\sum_{i=1}^{n}{\left(x_{i}^{0}-x_{i}^{f}\right)}^{2}}{n}$$

Extracts of the code in R for primary and secondary fittings are given in Supplementary Material (R-commands 1).

#### Correlations Between Growth Parameters

An analysis of covariance was performed to evaluate if the maximal bacterial growth rates (μ_*max*_) were significantly different between the two food packaging. All tests were considered as significant for a *p*-value of < 0.05. Extracts of the code in R for ANCOVA analysis are given in Supplementary Material (R-commands 2).

Using R software (R Core Team, 2019), correlations between the minimal shelf life (*MSL*) and the growth parameters *(*μ_*max*_, *LPD*, *N*_0_, *N*_*max*_) were obtained by the Pearson’s correlation coefficient (*r*) in mono-culture and co-culture experiments (Liu et al., 2006; Miks-Krajnik et al., 2016). High correlations were considered when |*r*| > 0.7000 (Miks-Krajnik et al., 2016). The best influencing growth parameter on the microbial shelf life was chosen according to the Pearson’s correlations coefficient.

Then, a reduction ratio (α) was calculated to quantify the interaction effect on μ_*max*_ by inoculated bacteria in co-culture experiments (Eq. 12) (Correia Peres Costa et al., 2019).

(12) $$\alpha =1-\frac{\left(p_{co}\right)}{\left(p_{mono}\right)}$$

Where α is the reduction ratio; *p*_*co*_ and *p*_*mono*_ are the growth parameters obtained in co-culture and mono-culture experiments, respectively.

#### Modeling Microbial Interactions for *B. thermosphacta*, *Pseudomonas* spp., and *L. gelidum*

Two well-known interactions models for two-species were modified to predict the simultaneous growth of the three-inoculated spoilage bacteria in irradiated MP samples: the modified Jameson-effect model and the Lotka Volterra model (Cornu et al., 2011; Correia Peres Costa et al., 2019).

As presented by Cornu et al. (2011) and Quinto et al. (2018), a modified generic primary growth model can be written as Eq. 13.

(13) $$\frac{1}{N\left(t\right)}\frac{dN\left(t\right)}{dt}=\frac{d\left(\ln\left(N\left(t\right)\right)\right)}{dt}=\mu_{max}\times \alpha \left(t\right)\times f\left(t\right)$$

Where $\frac{1}{N\left(t\right)}$$\frac{dN\left(t\right)}{dt}$ is the relative or instantaneous growth rate of the microorganism, *N*_*t*_ is the bacterial concentration at time *t* (log CFU/g), μ_*max*_ is the maximum growth rate (1/h), α*(t)* is an adjustment function, and *f(t)* is an inhibition function, defined as Eqs 14 and 15:

(14) $$\alpha_{t}=\left\{ \begin{array}{c} 0ift<LPD\\ 1ift\geq LPD \end{array}\right.$$

(15) $$f_{t}=\left(1-\left(\frac{N_{t}}{N_{max}}\right)\right)$$

Where *LPD* is the lag phase duration (hours) and *N*_*max*_ is the maximal population density (log CFU/g).

Based on Eq. 13, an alternative deceleration function can be added for modeling the interaction of two bacterial species (Jameson-effect model) (Eq. 16) (Mejlholm and Dalgaard, 2007; Cornu et al., 2011).

(16) $$\left\{ \begin{array}{c} \frac{1}{N_{A}\left(t\right)}\frac{dN_{A}\left(t\right)}{dt}=\mu_{maxA\left(t\right)}\times \alpha_{A}\left(t\right)\times \left(1-\frac{N_{A\left(t\right)}}{N_{maxA\left(t\right)}}\right)\\ \;\times \left(1-\frac{N_{B\left(t\right)}}{N_{maxB\left(t\right)}}\right)\\ \;\frac{1}{N_{B}\left(t\right)}\frac{dN_{Bt}}{dt}=\mu_{maxB\left(t\right)}\times \alpha_{B}\left(t\right)\times \left(1-\frac{N_{B\left(t\right)}}{N_{maxB\left(t\right)}}\right)\\ \;\times \left(1-\frac{N_{A\left(t\right)}}{N_{maxA\left(t\right)}}\right) \end{array}\right.$$

Where *N* is the cell concentration (log CFU/g) at time *t* (h), μ_*max*_ is the maximum specific growth rate (1/h), and *N*_*max*_ is the maximum population density (log CFU/g).

In the modified Jameson-effect model, the deceleration function can be replaced by Eq. 17 (Mejlholm and Dalgaard, 2007; Cornu et al., 2011; Quinto et al., 2018; Cadavez et al., 2019).

(17) $$\left\{ \begin{array}{c} f_{A}\left(t\right)=\left(1-\frac{N_{A}\left(t\right)}{N_{max_{A\left(t\right)}}}\right)\left(1-\frac{N_{B}\left(t\right)}{N_{max_{B\left(t\right)}}}\right)\\ f_{B}\left(t\right)=\left(1-\frac{N_{A}\left(t\right)}{N_{MCP_{A\left(t\right)}}}\right)\left(1-\frac{N_{B}\left(t\right)}{N_{max_{B}\left(t\right)}}\right)ifN_{A}\left(t\right)\geq N_{MCP_{A\left(t\right)}}\\ f_{B}\left(t\right)=0ifN_{A}\left(t\right)\geq N_{MCP_{A\left(t\right)}} \end{array}\right.$$

Where *N*_*t*_ is the bacterial concentration at time *t* (log CFU/g), *N*_*max*(t)_ is the maximal population density (log CFU/g), and *N*_*MCP(t)*_ is maximum critical population (log CFU/g) that the bacterium should be reached to inhibit the growth of the other populations. *MCP* is inferior to its own maximum population density (*N*_*max*_) (Cornu et al., 2011; Correia Peres Costa et al., 2019).

Using R software (R Core Team, 2019), the modified Jameson-effect model (Eq. 17) was applied on mono-culture experiment data with the functions of Baranyi, Buchanan and without-lag (package nlsMicrobio, Baty and Delignette-Muller, 2013). The function without lag shown the best fitting in all cases (Supplementary Table S1). This model was then selected in the rest of the study, by using the growth parameters obtained on co-culture experiments. Extracts of the code in R for the modified Jameson-effect models for two species are given in Supplementary Material (R-commands 3).

For a three-species mixed culture model, Quinto et al. (2018) recently proposed a modification of the logistic deceleration model (Eq. 18).

(18) $$f\left(t\right)=\left(1-\frac{N_{A}\left(t\right)+N_{B}\left(t\right)+N_{C}\left(t\right)}{N_{\max tot}}\right)$$

Where *N*_*A*_(*t*), *N*_*B*_(*t*), and *N*_*C*_(*t*) are the cell concentration of microorganism *A*, *B*, or *C* in co-culture at time *t*; *N*_*maxtot*_ is the maximal total population density (including all species present) and consequently the overall carrying capacity of the system from the three-species co-cultured.

However, this study only considers the effect of *P. fluorescens* and *L. innocua* on the bacterial growth of *L. monocytogenes*.

In our study, the aim of co-culture experiments was to consider the global effect of three inoculated bacterial species and the bacterial interaction on each other.

According to this, the modified Jameson-effect model was re-defined for a three-species model that was used in this study (Eq. 19).

(19) $$\begin{array}{c} \frac{1}{N_{tot}\left(t\right)}\frac{dN_{tot\left(t\right)}}{dt}=\mu_{max\left(Bm,Ps,Lg\right)\left(t\right)}\\ \;\times \alpha_{\left(Bm,Ps,Lg\right)}\left(t\right)\\ \;\times \left(1-\frac{N_{Bm\left(t\right)}+N_{Ps\left(t\right)}+N_{Lg\left(t\right)}}{N_{MCP}\left(t\right)}\right) \end{array}$$

Where *N* is the cell concentration (log CFU/g) at time *t* (h), μ_*max*_ is the maximum specific growth rate (1/h), α(*t*) is an adjustment function, and *N*_*MCP*_ is the maximum critical population of each bacterium (log CFU/g).

Extracts of the code in R for the three-species modified Jameson-effect models are given in Supplementary Material (R-commands 4).

In the two-species model based on the Lotka Volterra equation, the deceleration function can be replaced by Eq. 20 (Cornu et al., 2011), which includes empirical parameters reflecting the degree of interaction between microbial species (*F*_*AB*_ and *F*_*BA*_) (Liu et al., 2006; Cornu et al., 2011; Cadavez et al., 2019; Correia Peres Costa et al., 2019).

(20) $$\left\{ \begin{array}{c} f_{A}\left(t\right)=\left(1-\frac{N_{A}\left(t\right)+F_{AB}N_{B}\left(t\right)}{N_{\max A\left(t\right)}}\right)\\ f_{B}\left(t\right)=\left(1-\frac{N_{B}\left(t\right)+F_{BA}N_{A}\left(t\right)}{N_{\max B}\left(t\right)}\right) \end{array}\right.$$

Where the parameters *F*_*AB*_ and *F*_*BA*_ are the coefficients of interaction measuring the effects of one species on the other.

Using R software (R Core Team, 2019), the Lotka Volterra model (Eq. 20) was also re-defined for a three-species interaction model, represented by Eq. 21.

(21) $$\left\{ \begin{array}{cc} \frac{1}{N_{A}\left(t\right)}\frac{dN_{A\left(t\right)}}{dt}= & \mu_{maxA\left(t\right)}\times \alpha_{A}\left(t\right)\\ & \times \left(1-\frac{N_{A\left(t0\right)}+\left(F_{ABC}\times F_{ACB}\times N_{BC\left(t0\right)}\right)}{N_{maxA\left(t\right)}}\right)\\ \frac{1}{N_{B}\left(t\right)}\frac{dN_{B\left(t\right)}}{dt}= & \mu_{maxB\left(t\right)}\times \alpha_{B}\left(t\right)\\ & \times \left(1-\frac{N_{B\left(t0\right)}+\left(F_{BAC}\times F_{BCA}\times N_{AC\left(t0\right)}\right)}{N_{maxB\left(t\right)}}\right)\\ \frac{1}{N_{C}\left(t\right)}\frac{dN_{C\left(t\right)}}{dt}= & \mu_{maxC\left(t\right)}\times \alpha_{C}\left(t\right)\\ & \times \left(1-\frac{N_{C\left(t0\right)}+\left(F_{CAB}\times F_{CBA}\times N_{AB\left(t0\right)}\right)}{N_{maxC\left(t\right)}}\right) \end{array}\right.$$

Where *N* is the cell concentration (log CFU/g) at time *t* (h), μ_*max*_ is the maximum specific growth rate (1/h), α*(t)* is an adjustment function, *F*_*A,B,C*_ are the coefficient of interaction measuring the effects of one species on the others, and *N*_*max*_ is the maximum population density (log CFU/g).

Extracts of the code in R for the three-species Lotka Volterra models are given in Supplementary Material (R-commands 5).

Comparison of the two models was assessed by root-mean-square error (*RMSE*) and coefficient of determination (*R*^2^) (Correia Peres Costa et al., 2019), as previously described in the section above (Section 2.7.1.).

#### Model Validation

Validation of the developed three-species interaction models was performed using a new dataset of experimental data.

Fresh MP samples were obtained from a local Belgian manufacturer at the day of the production, corresponding to the day of slaughtering. MP samples were packed by the manufacturer in a polypropylene tray under cling film. Samples have the same composition as described above.

Samples were not irradiated and not inoculated in order to follow the dynamics of the natural food microbiota. MP samples were also packed (50 g) in two different packaging, in triplicate.

The first packaging was a tray (187 × 137 × 36, polyester 10 μm, homo-polymer polypropylene 50 μm, NutriPack, France) under modified atmosphere (MAP, CO_2_ 30%/O_2_ 70% ± 0.1%) (Olympia V/G, Technovac, Italy) using packaging wrap (PP/EVOH/PP) with random gas measurements (CheckMate 3, Dansensor, France). The second packaging consisted in a tray (175 × 135 × 22, polystyrene) under FW using cling film (Clinofilm).

In this study, MP samples were stored during a 13 days shelf life at isothermal temperature: (i) 4°C (±1°C), (ii) 8°C (±1°C), (iii) and 12°C (±1°C), in climatic chambers (Sanyo MIR 254).

Samples (*n* = 288) were then analyzed at the first day of inoculation (day 0) and daily until the last day of storage (day 13). Analyses were performed by classical plate counts and 16S rDNA metagenetics, as methods previously described in the sections above (sections “16S rDNA Metagenetic Approach” and “Approach Used to Develop the Interaction Model”), in order to estimate bacterial counts over the storage.

The performance of the developed interaction models was evaluated by the acceptable simulation zone (*ASZ*) approach. Model performance is considered acceptable when at least 70% of the observed log counts values are within the *ASZ*, defined as ± 0.5 log-units from the simulated concentration in log units (Correia Peres Costa et al., 2019).

## Results

### 16S rDNA Metagenetic Results

Despite of the inability of differentiation between viable and non-viable cells by the culture-independent DNA-based methods used, high level (>95%) of relative abundance for each inoculated bacterium was observed for mono-culture experiments (Supplementary Figure S2).

**FIGURE 2:**
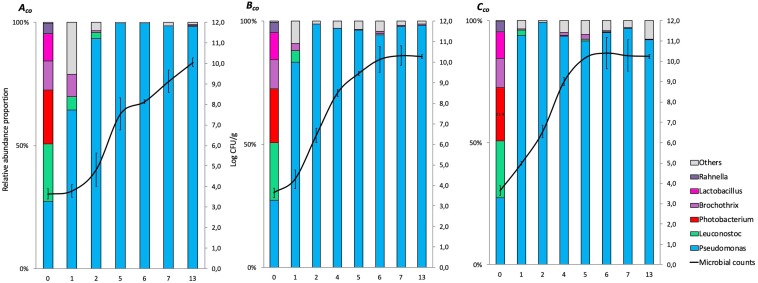
Cumulated histograms of the relative abundance (%) of taxa and the dynamics of the bacterial community identified by metagenetics at genus levels in co-culture experiment during storage in food wrap (***A***_*co*_, at 4°C; ***B***_*co*_, at 8°C; ***C***_*co*_, at 12°C). At genus levels, the taxa representing < 1% in relative abundance were merged in the category of “Others.” The solid represents the plate counts (means and standard deviation of the three replicates).

**FIGURE 3:**
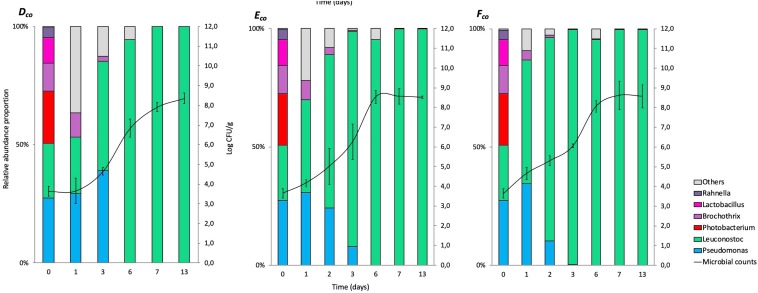
Cumulated histograms of the relative abundance (%) of taxa and the dynamics of the bacterial community identified by metagenetics at genus levels in co-culture experiment during storage in modified atmosphere packaging (***D***_*co*_, at 4°C; ***E***_*co*_, at 8°C; ***F***_*co*_, at 12°C). At genus levels, the taxa representing < 1% in relative abundance were merged in the category of “Others.” The solid represents the plate counts (means and standard deviation of the three replicates).

The relative abundance results for co-culture experiments (expressed in%) at genus levels (>1%) are represented in cumulated histograms for all samples in FW (Figure 2) and MAP (Figure 3). These data including the relative abundance of sequences are also summarized in Supplementary Table S2.

The taxa representing < 1% in relative abundance were merged in the category of “Others.” “Others” are mainly composed by the genera *Aeromonas*, *Arthrobacter*, *Bacteroides*, *Carnobacterium*, *Chryseobacterium*, *Enterococcus*, *Flavobacterium*, *Kurthia*, *Lactobacillus*, *Lactococcus*, *Mannheimia*, *Massilia*, *Micrococcus*, *Moraxella*, *Myroides*, *Ottowia*, *Peptococcus*, *Photobacterium*, *Porphyromonas*, *Propionibacterium*, *Rothia*, *Serratia*, and *Staphylococcus*. Full data on taxa found in high (>1%) and low (<1%) frequencies will be made available by EC to any qualified researcher.

At day 0, small differences between the distribution of read percentages for the three inoculated bacteria are observed (11.8, 27.4, and 23.3% for *Brochothrix*, *Pseudomonas*, and *Leuconostoc*, respectively).

At day 3 in FW, *Brochothrix* became under the detection limit. At this same time, *Pseudomonas* became the most represented genus (>90%), and remained during the 13 days of storage.

In MAP, *Leuconostoc* and *Pseudomonas* were equally distributed during the first days of storage, but *Leuconostoc* became the most represented genus (>90%) after 3 days and until the end of storage.

### Plate Counts and Estimated Abundance

In mono-culture experiments, plate counts for *B. thermosphacta*, *Pseudomonas* spp., and *L. gelidum* increased during the shelf life with increasing the temperature (Table 2).

**TABLE 2:** Microbiological counts (log CFU/g) for mono-culture experiments in minced pork samples stored during 13-days shelf life, at constant temperature, in food wrap (FW) and modified atmosphere packaging (MAP, CO_2_ 30%/O_2_ 70% ± 0.1%).

	Days													
Codes	0	1	2	3	4	5	6	7	8	9	10	11	12	13
*A*_*mono*_	3.84 ± 0.03	3.08 ± 0.10	3.76 ± 0.07	4.54 ± 0.12	–^a^	–^a^	7.24 ± 0.11	7.74 ± 0.17	7.63 ± 0.10	8.17 ± 0.33	7.68 ± 0.15	–^a^	–^a^	7.90 ± 0.15
*B*_*mono*_	3.84 ± 0.03	6.76 ± 0.04	7.49 ± 0.11	8.25 ± 0.07	8.51 ± 0.10	8.58 ± 0.06	8.85 ± 0.02	8.77 ± 0.15	9.05 ± 0.03	8.79 ± 0.21	–^a^	–^a^	–^a^	9.00 ± 0.01
*C*_*mono*_	3.84 ± 0.03	7.68 ± 0.08	8.29 ± 0.13	8.66 ± 0.04	8.99 ± 0.09	9.01 ± 0.23	9.11 ± 0.10	8.81 ± 0.28	9.03 ± 0.03	8.91 ± 0.16	–^a^	–^a^	–^a^	9.27 ± 0.08
*D*_*mono*_	3.84 ± 0.03	–^a^	–^a^	2.17 ± 0.30	–^a^	–^a^	4.11 ± 0.01	4.01 ± 0.14	4.35 ± 0.03	5.24 ± 0.05	4.99 ± 0.12	–^a^	–^a^	5.43 ± 0.06
*E*_*mono*_	3.84 ± 0.03	–^a^	5.88 ± 0.10	6.11 ± 0.11	7.11 ± 0.02	7.86 ± 0.10	8.21 ± 0.04	8.43 ± 0.11	8.43 ± 0.16	8.41 ± 0.10	8.38 ± 0.16	–^a^	7.86 ± 0.07	8.76 ± 0.03
*F*_*mono*_	3.84 ± 0.03	–^a^	7.10 ± 0.04	7.76 ± 0.23	8.35 ± 0.04	8.58 ± 0.06	8.40 ± 0.12	8.44 ± 0.07	8.32 ± 0.03	9.16 ± 0.08	8.67 ± 0.40	–^a^	8.83 ± 0.02	8.71 ± 0.06
*G*_*mono*_	3.15 ± 0.59	3.43 ± 0.11	4.52 ± 0.23	5.64 ± 0.19	–^a^	–^a^	–^a^	9.45 ± 0.13	9.51 ± 0.07	–^a^	9.90 ± 0.29	–^a^	–^a^	10.21 ± 0.03
*H*_*mono*_	3.15 ± 0.59	3.86 ± 0.17	5.36 ± 0.03	7.69 ± 0.17	9.04 ± 0.05	9.67 ± 0.03	–^a^	9.62 ± 0.15	10.34 ± 0.24	10.39 ± 0.40	10.11 ± 0.28	–^a^	–^a^	10.15 ± 0.17
*I*_*mono*_	3.15 ± 0.59	4.93 ± 0.15	–^a^	9.81 ± 0.04	9.85 ± 0.29	9.95 ± 0.34	10.15 ± 0.82	10.26 ± 0.08	10.14 ± 0.10	–^a^	9.87 ± 0.19	–^a^	–^a^	9.80 ± 0.42
*J*_*mono*_	3.15 ± 0.59	–^a^	3.48 ± 0.06	–^a^	–^a^	3.90 ± 0.11	4.87 ± 0.34	4.55 ± 0.12	–^a^	–^a^	–^a^	–^a^	4.73 ± 0.01	4.90 ± 0.01
*K*_*mono*_	3.15 ± 0.59	3.52 ± 0.01	4.16 ± 0.05	–^a^	–^a^	5.41 ± 0.08	6.33 ± 0.07	6.52 ± 0.14	–^a^	6.59 ± 0.17	–^a^	–^a^	7.83 ± 0.13	8.37 ± 0.08
*L*_*mono*_	3.15 ± 0.59	4.47 ± 0.07	6.08 ± 0.03	–^a^	–^a^	–^a^	9.42 ± 0.28	9.58 ± 0.23	–^a^	9.80 ± 0.41	–^a^	–^a^	9.87 ± 0.06	9.85 ± 0.14
*M*_*mono*_	4.00 ± 0.02	4.07 ± 0.01	4.38 ± 0.01	4.61 ± 0.12	–^a^	–^a^	6.17 ± 0.05	–^a^	–^a^	–^a^	8.62 ± 0.09	–^a^	–^a^	8.42 ± 0.06
*N*_*mono*_	4.00 ± 0.02	4.58 ± 0.08	5.84 ± 0.02	–^a^	7.57 ± 0.10	–^a^	8.61 ± 0.13	–^a^	8.73 ± 0.07	–^a^	8.84 ± 0.09	–^a^	–^a^	8.77 ± 0.30
*O*_*mono*_	4.00 ± 0.02	5.38 ± 0.01	6.84 ± 0.13	8.35 ± 0.09	7.56 ± 0.01	–^a^	8.64 ± 0.13	–^a^	–^a^	–^a^	8.82 ± 0.23	–^a^	–^a^	8.62 ± 0.18
*P*_*mono*_	4.00 ± 0.02	4.18 ± 0.09	–^a^	–^a^	6.31 ± 0.17	–^a^	6.84 ± 0.06	7.85 ± 0.01	–^a^	7.78 ± 0.21	–^a^	–^a^	8.00 ± 0.10	8.39 ± 0.12
*Q*_*mono*_	4.00 ± 0.02	4.75 ± 0.03	–^a^	–^a^	8.06 ± 0.01	–^a^	8.38 ± 0.05	8.49 ± 0.16	–^a^	8.85 ± 0.01	–^a^	–^a^	–^a^	8.75 ± 0.19
*R*_*mono*_	4.00 ± 0.02	8.32 ± 0.15	7.28 ± 0.01	–^a^	8.35 ± 0.06	–^a^	8.36 ± 0.09	8.64 ± 0.10	–^a^	8.89 ± 0.07	–^a^	–^a^	–^a^	8.87 ± 0.11

*See Table 1 for list of the codes used. Mean values with standard deviations of the three replicates. –^a^ no analysis performed for the day.*

At the end of the shelf life, the bacterial count was higher than 7.0 log CFU/g, except for some samples stored in MAP. During the storage, a high growth rate and a more rapidly reached stationary phase were also correlated to FW and the highest storage temperatures.

No bacterial growth was observed on PCA for the control samples (limit detection < 3.0 log CFU/g) (data not shown in this paper).

For co-culture experiments, the metagenetic data were combined with the plate counts results in order to obtain estimated bacterial counts (Table 3).

**TABLE 3:** Estimate bacterial counts for co-culture experiment.

	Time (days)								
Code	0	1	2	3	4	5	6	7	13
*A*_*co*(A)_	2.71 ± 0.24	2.75 ± 0.31	2.71 ± 0.81	–^a^	–^a^	–^a^	–^a^	–^a^	7.77 ± 0.20
*A*_*co(B)*_	3.07 ± 0.24	3.60 ± 0.31	4.80 ± 0.81	–^a^	–^a^	7.54 ± 0.77	8.14 ± 0.08	9.12 ± 0.53	10.04 ± 0.20
*A*_*co(C)*_	3.00 ± 0.24	2.52 ± 0.31	3.20 ± 0.81	–^a^	–^a^	4.54 ± 0.77	5.14 ± 0.08	5.79 ± 0.53	6.92 ± 0.20
*B*_*co(A)*_	2.71 ± 0.24	2.26 ± 0.31	–^a^	–^a^	–^a^	–^a^	7.13 ± 0.53	7.68 ± 0.20	8.00 ± 0.10
*B*_*co(B)*_	3.07 ± 0.24	4.23 ± 0.46	6.43 ± 0.34	–^a^	8.49 ± 0.18	9.43 ± 0.10	10.11 ± 0.64	10.31 ± 0.47	10.27 ± 0.10
*B*_*co(C)*_	3.00 ± 0.24	2.48 ± 0.31	1.70 ± 0.81	–^a^	–^a^	5.44 ± 0.08	6.61 ± 0.08	6.93 ± 0.20	7.15 ± 0.10
*C*_*co(A)*_	2.71 ± 0.24	2.58 ± 0.09	–^a^	–^a^	7.15 ± 0.20	8.46 ± 0.02	8.18 ± 0.77	7.58 ± 0.78	7.24 ± 0.10
*C*_*co(B)*_	3.07 ± 0.24	4.95 ± 0.09	6.55 ± 0.30	–^a^	8.97 ± 0.20	10.14 ± 0.02	10.38 ± 0.77	10.26 ± 0.78	10.21 ± 0.10
*C*_*co(C)*_	3.00 ± 0.24	3.32 ± 0.09	3.04 ± 0.30	–^a^	6.30 ± 0.20	8.02 ± 0.02	7.41 ± 0.77	7.10 ± 0.78	7.06 ± 0.10
*D*_*co(A)*_	2.71 ± 0.24	2.67 ± 0.64	–^a^	2.97 ± 0.19	–^a^	–^a^	3.83 ± 0.46	–^a^	3.83 ± 0.46
*D*_*co(B)*_	3.07 ± 0.24	3.13 ± 0.64	–^a^	4.24 ± 0.19	–^a^	–^a^	4.14 ± 0.46	5.28 ± 0.23	4.76 ± 0.28
*D*_*co(C)*_	3.00 ± 0.24	3.04 ± 0.64	–^a^	4.31 ± 0.19	–^a^	–^a^	6.81 ± 0.46	7.91 ± 0.23	8.36 ± 0.28
*E*_*co(A)*_	2.71 ± 0.24	3.07 ± 0.19	3.46 ± 0.90	3.95 ± 0.90	–^a^	–^a^	–^a^	–^a^	4.94 ± 0.07
*E*_*co(B)*_	3.07 ± 0.24	3.65 ± 0.19	4.39 ± 0.90	5.15 ± 0.90	–^a^	–^a^	–^a^	5.00 ± 0.39	4.94 ± 0.07
*E*_*co(C)*_	3.00 ± 0.24	3.76 ± 0.19	4.82 ± 0.90	6.21 ± 0.90	–^a^	–^a^	8.51 ± 0.33	8.56 ± 0.39	8.50 ± 0.07
*F*_*co(A)*_	2.71 ± 0.24	3.25 ± 0.30	3.30 ± 0.25	–^a^	–^a^	–^a^	5.05 ± 0.30	5.51 ± 0.72	5.88 ± 0.58
*F*_*co(B)*_	3.07 ± 0.24	4.20 ± 0.30	4.31 ± 0.25	3.34 ± 0.10	–^a^	–^a^	–^a^	5.63 ± 0.72	4.98 ± 0.58
*F*_*co(C)*_	3.00 ± 0.24	4.38 ± 0.30	5.24 ± 0.25	6.05 ± 0.10	–^a^	–^a^	8.03 ± 0.30	8.61 ± 0.72	8.57 ± 0.58

*See Table 1 for list of the codes used. Mean values with standard deviations of the three replicates. FW, food wrap; MAP, modified atmosphere packaging (CO_2_ 30%/O_2_ 70% ± 0.1%), –^a^ no analysis performed for the day.*

As previously observed, estimate counts increased during the shelf life with increasing the temperature. At the end of the shelf life, the bacterial count was over 7.0 log CFU/g, except for *B. thermosphacta* and *Pseudomonas* spp. stored in MAP. During the storage, the same growth profiles as mono-culture experiments were observed.

### pH and Gas Measurements

A significant increase of pH is observed for MP samples inoculated by *Pseudomonas* spp. (7.54 ± 0.76, *n* = 5, *p*-value = 0.01) compared to the control samples (5.79 ± 0.05, *n* = 10).

In co-culture experiments, pH values at the end of the shelf life were not different to control samples (5.87 ± 0.02, *n* = 5) (Supplementary Figure S3).

A relatively stable concentration of carbon dioxide was observed in MAP at the end of the shelf life. Except for MP samples inoculated with *Pseudomonas* spp., which reached a higher significant carbon dioxide value (100.0 ± 0.1%) at 12°C (Supplementary Figure S4).

### Microbial Growth Parameters

Results of the primary and secondary model fittings for mono- and co-culture experiments are shown in Tables 4, 5. Growth parameters from mono-culture experiments are based on plate counts, and those from co-culture experiments are based on estimate abundance (obtained by the association of metagenetic and plate counts results).

**TABLE 4:** Observed kinetic parameters of mono- and co-culture experiments, calculated by Baranyi equation without interactions.

	μ_*max*_	*LPD*	*N*_0_	*N*_*max*_	*RSS*	*S*_*val*_	*MSL*
*A*_*mono*_	0.09[0.09–0.08]	51[53–51]	3.84 ± 0.03	7.90 ± 0.15	0.000442	Y	5.7[5.8–5.6]
*B*_*mono*_	0.21[0.21–0.19]	0[0–0]	3.84 ± 0.03	8.79 ± 0.21	0.000255	Y	1.5[1.5–1.4]
*C*_*mono*_	0.39[0.39–0.35]	0[0–0]	3.84 ± 0.03	9.11 ± 0.10	0.000558	Y	0.8[0.8–0.8]
*D*_*mono*_	0.03[0.03–0.03]	20[20–17]	3.84 ± 0.03	4.99 ± 0.12	0.005700	N	15.3[15.8–14.7]
*E*_*mono*_	0.07[0.07–0.07]	0[0–0]	3.84 ± 0.03	8.43 ± 0.16	0.005700	Y	3.8[3.9–3.7]
*F*_*mono*_	0.13[0.13–0.12]	0[0–0]	3.84 ± 0.03	8.83 ± 0.16	0.005260	Y	1.9[1.9–1.4]
*G*_*mono*_	0.06[0.06–0.06]	24[24–24]	3.15 ± 0.59	9.90 ± 0.29	0.010900	Y	4.5[4.6–4.2]
*H*_*mono*_	0.13[0.13–0.13]	10[10–10]	3.15 ± 0.59	10.15 ± 0.17	0.010900	Y	2.7[2.8–2.6]
*I*_*mono*_	0.23[0.23–0.23]	0[0–0]	3.15 ± 0.59	9.95 ± 0.34	0.010900	Y	1.8[1.9–1.7]
*J*_*mono*_	0.04[0.04–0.04]	48[48–48]	3.15 ± 0.59	4.90 ± 0.01	0.001210	N	21.8[22.6–20.9]
*K*_*mono*_	0.08[0.08–0.08]	27[27–27]	3.15 ± 0.59	8.37 ± 0.08	0.001210	Y	9.0[9.2–8.8]
*L*_*mono*_	0.13[0.13–0.13]	0[0–0]	3.15 ± 0.59	9.87 ± 0.06	0.001210	Y	3.5[3.6–3.3]
*M*_*mono*_	0.01[0.01–0.01]	48[48–48]	4.00 ± 0.02	8.42 ± 0.06	0.017900	Y	7.1[7.2–7.0]
*N*_*mono*_	0.07[0.08–0.07]	10[12–10]	4.00 ± 0.02	8.77 ± 0.30	0.023000	Y	3.4[3.4–3.3]
*O*_*mono*_	0.18[0.19–0.18]	0[0–0]	4.00 ± 0.02	8.64 ± 0.13	0.017900	Y	2.5[2.5–2.4]
*P*_*mono*_	0.02[0.02–0.02]	17[19–15]	4.00 ± 0.02	8.00 ± 0.10	0.025600	Y	6.2[6.4–5.5]
*Q*_*mono*_	0.13[0.13–0.13]	0[0–0]	4.00 ± 0.02	8.75 ± 0.19	0.023700	Y	3.0[3.0–2.3]
*R*_*mono*_	0.32[0.33–0.32]	0[0–0]	4.00 ± 0.02	8.87 ± 0.11	0.025600	Y	1.2[1.2–1.1]
*A*_*co*(A)_	0.03[0.03–0.03]	36[36–36]	2.71 ± 0.24	7.77 ± 0.20	0.000490	Y	11.2[11.6–10.6]
*A*_*co(B)*_	0.05[0.06–0.05]	12[12–12]	3.07 ± 0.24	10.04 ± 0.20	0.098240	Y	5.4[6.1–4.8]
*A*_*co(C)*_	0.01[0.01–0.01]	24[30–24]	3.00 ± 0.24	6.92 ± 0.20	0.002650	N	11.6[12.3–10.6]
*B*_*co(A)*_	0.07[0.08–0.07]	12[12–12]	2.71 ± 0.24	8.00 ± 0.10	0.014000	Y	7.8[8.3–7.3]
*B*_*co(B)*_	0.11[0.12–0.11]	0[0–0]	3.07 ± 0.24	10.27 ± 0.20	0.472000	Y	3.8[4.2–3.5]
*B*_*co(C)*_	0.05[0.05–0.05]	24[24–24]	3.00 ± 0.24	7.15 ± 0.10	0.016460	Y	8.5[8.8–8.2]
*C*_*co(A)*_	0.13[0.15–0.12]	0[0–0]	2.71 ± 0.24	7.58 ± 0.92	0.117000	Y	6.0[6.4–5.6]
*C*_*co(B)*_	0.19[0.20–0.19]	0[0–0]	3.07 ± 0.24	10.26 ± 0.78	0.472000	Y	3.5[3.9–3.3]
*C*_*co(C)*_	0.12[0.13–0.11]	0[0–0]	3.00 ± 0.24	7.10 ± 0.90	0.000840	Y	6.6[7.1–6.1]
*D*_*co(A)*_	0.02[0.02–0.01]	46[59–10]	2.71 ± 0.24	3.83 ± 0.46	0.000150	N	21.0[20.5–16.8]
*D*_*co(B)*_	0.06[0.06–0.03]	48[48–48]	3.07 ± 0.24	4.76 ± 0.75	0.135300	N	17.2[17.4–16.9]
*D*_*co(C)*_	0.01[0.02–0.01]	12[12–12]	3.00 ± 0.24	8.36 ± 0.28	0.046870	Y	7.6[8.2–7.0]
*E*_*co(A)*_	0.04[0.06–0.03]	16[16–16]	2.71 ± 0.24	4.94 ± 0.07	0.005560	N	23.1[24.0–15.6]
*E*_*co(B)*_	0.12[0.12–0.07]	16[16–16]	3.07 ± 0.24	5.00 ± 0.40	0.059240	N	14.4[21.2–8.5]
*E*_*co(C)*_	0.08[0.08–0.07]	6[6–6]	3.00 ± 0.24	8.50 ± 0.45	0.076910	Y	5.9[6.6–5.1]
*F*_*co(A)*_	0.07[0.10–0.06]	0[0–0]	2.71 ± 0.24	5.88 ± 0.01	0.006320	N	14.0[16.7–11.8]
*F*_*co(B)*_	0.20[0.21–0.12]	0[0–0]	3.07 ± 0.24	5.00 ± 0.56	0.015400	N	14.0[17.5–11.3]
*F*_*co(C)*_	0.20[0.20–0.16]	0[0–0]	3.00 ± 0.24	8.57 ± 0.73	0.030760	Y	5.9[6.6–5.2]

*See Table 1 for list of the codes used. Mean values with standard deviation (SD represent three samples per experiment) or with the 95% confidence intervals (lower limit and upper limit); μ_max_, maximal specific growth rate (1/h); LPD, lag phase duration (h); N_0_, initial bacterial concentration (log CFU/g); N_max_, maximum bacterial concentration (log CFU/g); RSS, residual sum of square of the model; S_val_, spoilage values of 7.00 log CFU/g [Y (yes) or N(not) if this value is reached during the 13-days shelf life]; MSL, predictions of the minimal shelf life for the product (days).*

**TABLE 5:** Estimation of the secondary parameters obtained by the square root model without interactions.

Mono-culture experiments		*T*_*min*_	Adjusted *T*_*min*_	μ_*ref*_	*RSS*
FW	*B. thermosphacta*	–3.36	−3.36	0.99 [0.99–0.89]	0.000668
FW	*Pseudomonas* spp.	–5.00	−5.02	0.42 [0.42–0.42]	0.001070
FW	*Ln. gelidum*	+ 1.00	+1.40	0.39 [0.41–0.39]	0.004580
MAP	*B. thermosphacta*	–3.36	−3.36	0.33 [0.33–0.32]	0.000003
MAP	*Pseudomonas* spp.	–5.00	−5.02	0.24 [0.24–0.24]	0.000323
MAP	*Ln. gelidum*	+ 1.00	+1.40	0.71 [0.73–0.71]	0.000033
**Co-culture experiments**					
FW	*B. thermosphacta*	–3.36	–^a^	0.30 [0.35–0.28]	0.000193
FW	*Pseudomonas* spp.	–5.02	–^a^	0.42 [0.44–0.42]	0.000190
FW	*Ln. gelidum*	+ 1.40	–^a^	0.35 [0.40–0.34]	0.000008
MAP	*B. thermosphacta*	–3.36	–^a^	0.17 [0.24–0.13]	0.000092
MAP	*Pseudomonas* spp.	–5.02	–^a^	0.43 [0.46–0.27]	0.023100
MAP	*Ln. gelidum*	+ 1.40	–^a^	0.59 [0.61–0.49]	0.000750

*Mean values with the 95% confidence intervals (lower limit and upper limit). FW, food wrap; MAP, modified atmosphere packaging (CO_2_ 30%/O_2_ 70% ± 0.1%); –^a^ not calculated in the model; T_min_, minimal temperature for growth (°C) provided from scientific literature; Adjusted T_min_, minimal temperature for growth (°C) provided from adjustment by the Rosso model (°C); μ_ref_, bacterial growth rate at the reference (1/h) obtained using a reparameterized version of the square root secondary model; RSS, residual sum of square for the μ_ref_ value.*

Good fit indexes were obtained in all cases (Supplementary Tables S3, S4).

Growth parameters showed different dynamic changes depending on storage temperature: a high storage temperature is correlated to a high growth rate during exponential phase and a lower lag-time. These growth parameters are also higher in FW than in MAP.

The *MSL* value is more rapidly reached in FW, except for *L. gelidum*.

Moreover, the *S*_*val*_ was never reached in MAP for MP samples inoculated by *Pseudomonas* spp. and *B. thermosphacta* during the 13-days shelf-life at 4°C.

**FIGURE 4:**
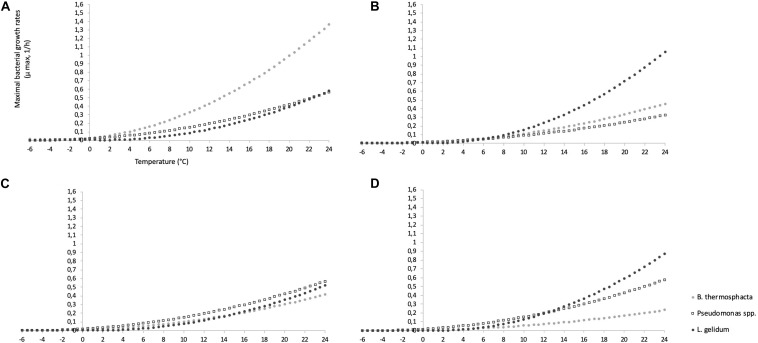
Evolution of μ_*max*_ between a large range of temperature (from −6 to + 25°C) for mono-culture experiments in FW **(A)** and MAP **(B)**, and for co-culture experiments in FW **(C)** and MAP **(D)**.

Based on these results, the evolution of μ_*max*_ between a large range of temperature (from −6 to +25°C) in FW and MAP was performed for mono- and co-culture experiments (Figure 4).

It can be clearly observed that *L. gelidum* had a highest growth rate in MAP, while it concerns *B. thermosphacta* in FW in mono-culture experiments. *B. thermosphacta* had the lowest one in co-culture experiments.

### Correlations Between Growth Parameters Obtained in Mono- and Co-culture Experiments

Correlations between growth parameters of *B. thermosphacta*, *Pseudomonas* spp., and *L. gelidum* for mono-culture and co-culture experiments are presented in Table 6.

**TABLE 6:** Correlations between growth parameters and the minimal shelf life (*MSL*) for mono-culture and co-culture experiments.

		Mono-culture experiments			Co-culture experiments		
Bacterial species/packaging	Growth parameters	Pearsons-correlations (*r*)	CI	*p*-value	Pearsons-correlations (*r*)	CI	*p*-value
**FW**							
*B. thermosphacta*	*μ*_*max*_	–0.8660	−0.9715;−0.4771	0.0025	–0.9144	−0.9821;−0.6376	0.0005
	*LPD*	0.9920	0.9608;0.9983	1.52^–07^	0.9839	0.9227;0.9967	1.71^–06^
	*N*_0_	0.0188	−0.6534;0.6745	0.9617	0.1763	−0.5524;0.7523	0.6500
	*N*_*max*_	–0.9553	−0.9908;−0.7965	5.94^–05^	0.2151	−0.5238;0.7693	0.5783
*Pseudomonas* spp.	*μ*_*max*_	–0.9548	−0.9907;−0.7945	6.17^–05^	–0.7774	−0.9507;−0.2344	0.0136
	*LPD*	0.9905	0.9542;0.9980	2.63^–07^	0.9013	0.5911;0.9792	0.0008
	*N*_0_	0.9903	−0.6048;0.7160	0.7999	0.3903	−0.3696;0.8373	0.2990
	*N*_*max*_	–0.0675	−0.7002;0.6245	0.8629	0.0278	−0.6482;0.6783	0.9434
*L. gelidum*	*μ*_*max*_	–0.8784	−0.9742;−0.5144	0.0018	–0.9160	−0.9824;−0.6434	0.0005
	*LPD*	0.9989	0.9948;0.9997	1.23^–10^	0.8251	0.3563;0.9620	0.0061
	*N*_0_	0.0271	−0.6486;0.6790	0.9448	0.2163	−0.5228;0.7698	0.5760
	*N*_*max*_	–0.5478	−0.8886;0.1828	0.1268	–0.0568	−0.6947;0.6311	0.8846
** MAP**							
*B. thermosphacta*	*μ*_*max*_	–0.8819	−0.9750;−0.5258	0.0016	–0.2501	−0.7839;0.4965	0.5164
	*LPD*	0.9881	0.9424;0.9975	5.95^–07^	0.5490	−0.1811;0.8890	0.1257
	*N*_0_	0.0411	−0.6405;0.6864	0.9164	0.5858	−0.1281;0.8998	0.0973
	*N*_*max*_	–0.9925	−0.9984;−0.9637	1.15^–07^	–0.4274	−0.8502;0.3304	0.2511
*Pseudomonas* spp.	*μ*_*max*_	–0.9572	−0.9912;−0.8047	5.09^–05^	–0.0339	−0.6827;0.6446	0.9308
	*LPD*	0.9549	0.7951;0.9907	6.10^–05^	0.3844	−0.3755;0.8352	0.3070
	*N*_0_	0.0425	−0.6396;0.6872	0.9134	0.7422	0.1540;0.9420	0.2202
	*N*_*max*_	–0.9977	−0.9995;−0.9890	1.66^–09^	0.2979	−0.4565;0.8031	0.4362
*L. gelidum*	*μ*_*max*_	–0.9283	−0.9851;−0.6891	0.0003	–0.5587	−0.8919;0.1675	0.1178
	*LPD*	0.9424	0.7438;0.9881	0.0001	0.7049	0.0768;0.9325	0.0339
	*N*_0_	0.1130	−0.5958;0.7228	0.7722	0.5667	−0.1561;0.8942	0.1116
	*N*_*max*_	–0.8983	−0.9786;−0.5806	0.0009	0.3732	−0.3867;0.8313	0.3225

*N_0_, the initial bacterial population (log CFU/g); N_max_, the maximal bacterial population (log CFU/g); LPD, the lag phase duration (h), μ_max_ (the maximum specific growth rate (1/h).*

It can be observed that the maximum specific growth rate (μ_*max*_) of micro-organisms was negatively correlated with microbial shelf life. The correlation was higher in mono-culture (−0.8660 to −0.9572) than in co-culture experiments (−0.0339 to −0.9160).

Lag phase duration (*LPD*) of all micro-organisms showed good correlation. High correlations of μ_*max*_ and *LPD* were observed in FW for co-culture experiments.

*N*_0_ showed little correlations than the two others parameters, except for mono-culture of *Pseudomonas* spp. stored in FW.

Moreover, no obvious correlation has been shown between *N*_*max*_ with shelf life for co-cultures experiments.

In conclusion, the results showed in our study that the microbial shelf life of MP samples is mainly correlated with μ_*max*_ and *LPD* than by *N*_*max*_ and *N*_0_. Even if the correlations are lower for experiments carried out in co-culture under MAP.

**FIGURE 5:**
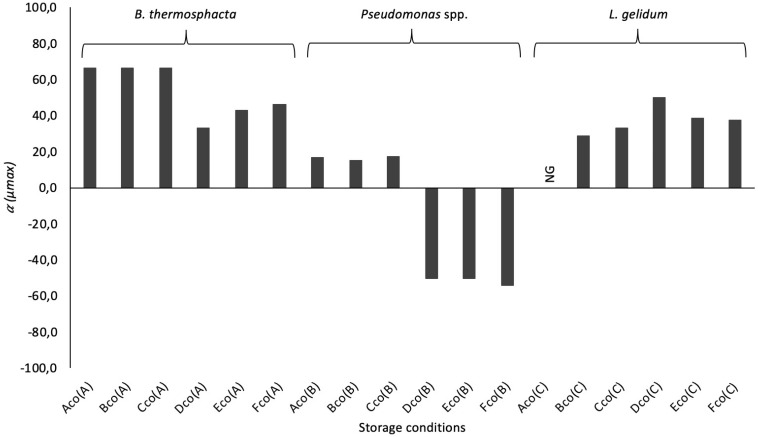
Reduction ratio (α), in%, of the parameters μ_*max*_ for *B. thermosphacta*, *Pseudomonas* spp., and *L. gelidum* in co-culture experiments at different storage conditions (see Table 1 for legend). The negative bars represent an increase in co-culture for the specific parameters. No growth of bacteria (NG) was only observed for *Ln. gelidum* in MAP at 4°C.

It was also showed that μ_*max*_ seems to be mainly influenced by the food packaging (Table 7), and by the interaction of the storage conditions applied in this study (packaging and temperature). These results were confirmed by the study of the reduction ratio α (Figure 5). *B. thermosphacta* and *L. gelidum* presented a higher reduction in FW. But an increase was observed for *Pseudomonas* spp. in MAP. Indeed, μ_*max*_ of *Pseudomonas* spp. was 0.04, 0.08, and 0.13, at 4, 8, and 12°C, respectively, in mono-culture experiments. While the parameter was gradually increasing to 0.06 (α = −50.0%), 0.12 (α = −50.0%), and 0.20 (α = −53.8%), at 4, 8, and 12°C, respectively, in co-culture experiments. However, *N*_*max*_ values of this bacterium were lesser in co-culture than in mono-culture experiments.

**TABLE 7:** Effect of food storage conditions on the maximal bacterial growth rates (μ_*max*_, 1/h) for mono- and co-cultures experiments (analysis of covariance, ANCOVA).

	Effects		
Experiments	Packaging	Temperature	Packaging * temperature^a^
**Mono-culture**			
*B. thermosphacta*	0.0113*	0.0003*	0.0001*
*Pseudomonas* spp.	0.4133	0.7389	0.0050*
*L. gelidum*	0.1655	0.0015*	0.4331
** Co-culture**			
*B. thermosphacta*	0.0280*	0.8072	0.0016*
*Pseudomonas* spp.	0.3063	0.3564	0.8114
*L. gelidum*	0.1030	0.1691	0.8728

^a^Interaction effect of packaging and temperature on bacterial growth rates; *significant statistical effect (p < 0.05).

### Three-Species Interaction Models and Validation Step

Estimated growth parameters and goodness-of-fit indexes for the two developed interaction models are available in Table 8.

**TABLE 8:** Estimated growth parameters of the three-species modified Jameson-effect and Lotka Volterra models, with goodness-of-fit indexes.

	Modified Jameson-effect model		Lotka Volterra model						
	*RrMSE*	μ_*max*_	*RrMSE*	*F*_*ABC*_	*F*_*ACB*_	*F*_*BAC*_	*F*_*BCA*_	*F*_*CAB*_	*F*_*CBA*_
*A*_*co*(A)_	0.261	0.047 [0.019; 0.076]	0.154	−0.90[−5.41;−0.19]	−1.10[−5.13;−0.18]	2.20 [0.92; 2.81]	0.45 [0.35; 1.08]	0.50[0.19;1.82]	1.99[0.54;5.00]
*A*_*co(B)*_	0.273	0.065 [0.031; 0.097]	0.171	−0.90[−5.41;−0.19]	−1.10[−5.13;−0.18]	2.20 [0.92; 2.81]	0.45 [0.35; 1.08]	0.50[0.19;1.82]	1.99[0.54;5.00]
*A*_*co(C)*_	0.284	0.039 [0.013; 0.065]	0.199	−0.90[−5.41;−0.19]	−1.10[−5.13;−0.18]	2.20 [0.92; 2.81]	0.45 [0.35; 1.08]	0.50[0.19;1.82]	1.99[0.54;5.00]
*B*_*co(A)*_	0.372	0.230 [0.019; 0.380]	0.113	0.05[−0.02;0.09]	6.02[3.53;6.55]	0.90 [0.85; 0.99]	1.08 [0.67; 1.11]	−5.51[−5.73;−0.27]	−0.04[−0.05;−0.03]
*B*_*co(B)*_	0.273	0.317 [0.031; 0.485]	0.365	0.05[−0.02;0.09]	6.02[3.53;6.55]	0.90 [0.85; 0.99]	1.08 [0.67; 1.11]	−5.51[−5.73;−0.27]	−0.04[−0.05;−0.03]
*B*_*co(C)*_	0.284	0.184 [0.015; 0.327]	0.108	0.05[−0.02;0.09]	6.02[3.53;6.55]	0.90 [0.85; 0.99]	1.08 [0.67; 1.11]	−5.51[−5.73;−0.27]	−0.04[−0.05;−0.03]
*C*_*co(A)*_	0.224	0.111 [0.082; 0.140]	0.216	0.11[0.04;0.17]	0.38[0.17;0.50]	0.62 [0.61; 0.63]	1.15 [1.06; 1.21]	0.78[0.60;1.06]	0.12[0.12;0.15]
*C*_*co(B)*_	0.248	0.136 [0.105; 0.169]	0.294	0.11[0.04;0.17]	0.38[0.17;0.50]	0.62 [0.61; 0.63]	1.15 [1.06; 1.21]	0.78[0.60;1.06]	0.12[0.12;0.15]
*C*_*co(C)*_	0.250	0.090 [0.062; 0.116]	0.186	0.11[0.04;0.17]	0.38[0.17;0.50]	0.62 [0.61; 0.63]	1.15 [1.06; 1.21]	0.78[0.60;1.06]	0.12[0.12;0.15]
*D*_*co(A)*_	0.187	0.015 [0.004; 0.028]	0.056	−0.06[−0.14;0.15]	−11.08[−11.08;−3.72]	2.21 [1.80; 2.21]	0.45 [0.45; 0.48]	−5.05[−5.05;0.50]	0.13[−0.32;0.37]
*D*_*co(B)*_	0.186	0.018 [0.004; 0.033]	0.205	−0.06[−0.14;0.15]	−11.08[−11.08;−3.72]	2.21 [1.80; 2.21]	0.45 [0.45; 0.48]	−5.05[−5.05;0.50]	0.13[−0.32;0.37]
*D*_*co(C)*_	0.223	0.064 [0.004; 0.084]	0.083	−0.06[−0.14;0.15]	−11.08[−11.08;−3.72]	2.21 [1.80; 2.21]	0.45 [0.45; 0.48]	−5.05[−5.05;0.50]	0.13[−0.32;0.37]
*E*_*co(A)*_	0.187	0.044 [0.023; 0.095]	0.050	0.26[−0.24;0.26]	3.08[−3.96;3.08]	4.40 [1.31; 4.40]	0.14 [0.11; 0.75]	−0.28[−0.28;3.01]	−0.74[−0.74;0.32]
*E*_*co(B)*_	0.228	0.039 [0.014; 0.096]	0.094	0.26[−0.24;0.26]	3.08[−3.96;3.08]	4.40 [1.31; 4.40]	0.14 [0.11; 0.75]	−0.28[−0.28;3.01]	−0.74[−0.74;0.32]
*E*_*co(C)*_	0.186	0.110 [0.055; 0.184]	0.119	0.26[−0.24;0.26]	3.08[−3.96;3.08]	4.40 [1.31; 4.40]	0.14 [0.11; 0.75]	−0.28[−0.28;3.01]	−0.74[−0.74;0.32]
*F*_*co(A)*_	0.192	0.056 [0.015; 0.095]	0.203	−0.15[−0.19;0.02]	−0.11[−0.20;0.01]	0.66 [0.40; 0.83]	0.47 [0.43; 0.48]	0.63[0.60;0.63]	1.19[1.19;1.27]
*F*_*co(B)*_	0.228	0.035 [0.010; 0.096]	0.189	−0.15[−0.19;0.02]	−0.11[−0.20;0.01]	0.66 [0.40; 0.83]	0.47 [0.43; 0.48]	0.63[0.60;0.63]	1.19[1.19;1.27]
*F*_*co(C)*_	0.186	0.100 [0.046; 0.184]	0.221	−0.15[−0.19;0.02]	−0.11[−0.20;0.01]	0.66 [0.40; 0.83]	0.47 [0.43; 0.48]	0.63[0.60;0.63]	1.19[1.19;1.27]

*See Table 1 for the list of codes used. Mean values with the 95% confidence intervals (lower limit and upper limit). RrMSE, the root-mean-square error of the residuals; μ_max_, the maximum growth rate (1/h); F_ABC_, F_ACB_, F_BAC_, F_BCA_, F_CAB_, F_CBA_, the coefficient of interaction measuring the effects of one species on the others (_A_, B. thermosphacta; _B_, Pseudomonas spp.; _C_, L. gelidum; respectively).*

The Lotka Volterra model showed lower *RrMSE* values but the interaction factors are sometimes included in high intervals.

**FIGURE 6:**
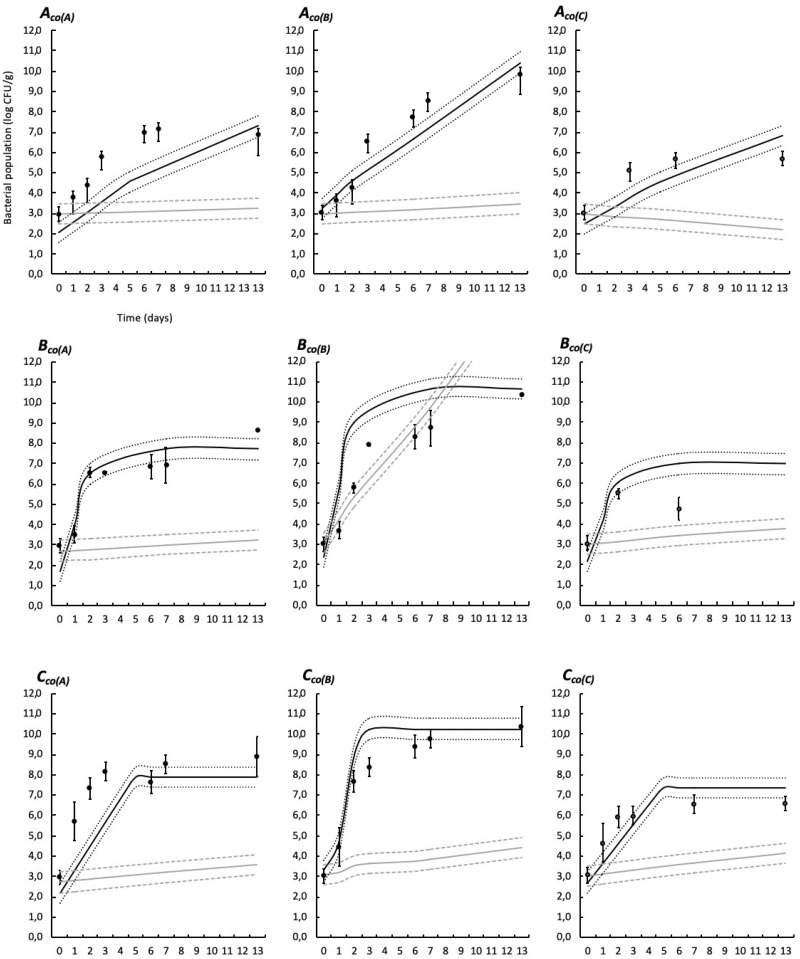
Experimental observed data (validation dataset, means, and standard deviation of the three replicates) and simulations provided by the predictive models based on the modified Jameson-effect equation and on the Lotka Volterra equation in food wrap. See Table 1 for list of the codes used. Black solid lines represent the Jameson-effect model and gray solid lines represent the Lotka Volterra model. Dashed and dotted lines represent the acceptable simulation zone (ASZ) used to compare observations versus predictions of the interaction models.

**FIGURE 7:**
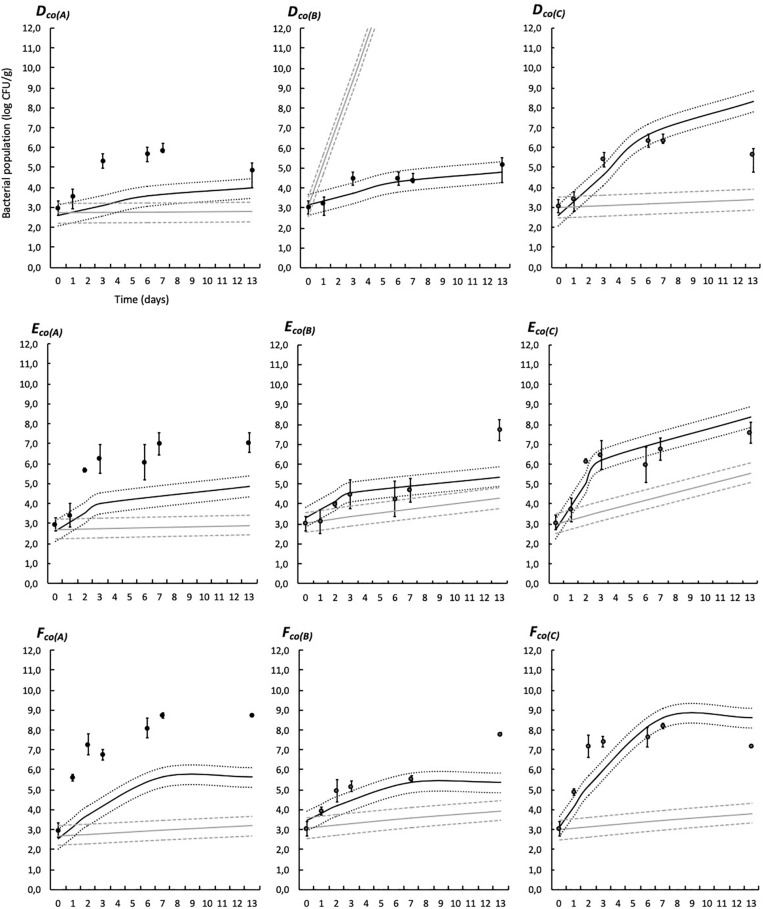
Experimental observed data (validation dataset, means, and standard deviation of the three replicates) and simulations provided by the predictive models based on the modified Jameson-effect equation and on the Lotka Volterra equation in modified atmosphere packaging. See Table 1 for list of the codes used. Black solid lines represent the Jameson-effect model and gray solid lines represent the Lotka Volterra model. Dashed and dotted lines represent the acceptable simulation zone (ASZ) used to compare observations versus predictions of the interaction models.

Simulations provided by the predictive models based on the modified Jameson-effect model and the Lotka Volterra equations are represented in Figures 6, 7.

The modified Jameson-effect model showed the best model performance (*ASZ*), with a mean of 63 ± 23%, while the Lotka Volterra model showed lesser percentages [31 ± 17% (*n* = 18)]. Eight simulated models based on the equation of the modified Jameson-effect model can be considered as acceptable, because at least 70% of the observed log counts values are within the *ASZ*.

### Validation Dataset

As previously described, plate counts in validation dataset increased during the shelf life with increasing the temperature (Supplementary Figures S5, S6).

At the end of the shelf life, the natural logarithm of the bacterial count was over 7.0 log CFU/g.

During the storage, a high growth rate and a more rapidly reached stationary phase are also correlated to FW and the highest storage temperatures.

No bacterial growth was observed on PCA for the control samples (limit detection < 3.00 log CFU/g) (data not shown in this paper).

The relative abundance results obtained by metagenetic analysis (expressed in%) at species levels (>1%) are represented in cumulated histograms for validation dataset in Supplementary Material for FW (Supplementary Table S5) and MAP (Supplementary Table S6). The metagenetic data were then combined with the plate counts results in order to obtain estimated bacterial counts (Supplementary Table S7).

At day 0, the distribution of read percentages shows high values (>90%) of *Photobacterium* spp., *Photobacterium kishitanii* and *Photobacterium illiopiscarium*.

In FW, *Pseudomonas* spp. reached higher values at day 3, and became the most represented bacteria until the end of the shelf-life (>90%). *B. thermosphacta* reached lesser values, with 3.22% at the end of the shelf-life. *L. gelidum* was always under the detection limit. These results are in accordance with those obtained in co-culture experiments.

In MAP, *Photobacterium* spp. was the most represented genus (>90%) during storage. However, low levels of *B. thermosphacta* and *L. gelidum* were observed at 8 and 12°C. *Pseudomonas* spp. was always under the detection limit. These results are different from those obtained in co-culture experiments.

Moreover, pH value of the validation dataset at the end of the shelf-life was statistically different to control samples (7.06 ± 0.80, *n* = 7, *p*-value = 0.01).

At the same time, the concentration of carbon dioxide also showed higher values than control samples (35.5 ± 1.64, 56.7 ± 2.17, and 96.7 ± 5.57, at 4, 8, and 12°C, respectively).

## Discussion

The present study aimed to obtain the growth parameters of three specific spoilage micro-organisms previously isolated in MP samples, and to develop a three-spoilage species interaction model under different storage conditions. *B. thermosphacta*, *Pseudomonas* spp., and *L. gelidum* were previously isolated as predominant strains (>50% reads) from different batches of Belgian MP samples at the end of their use-by-date (Cauchie et al., 2019). Considered as the main representative spoilage species in meat and meat products (Koort et al., 2005; Liu et al., 2006; Nychas et al., 2008; Pennacchia et al., 2009, 2011; Andritsos et al., 2012; De Filippis et al., 2013; Casaburi et al., 2014; Stoops et al., 2015; Zhao et al., 2015; Mann et al., 2016; Stellato et al., 2016; Del Blanco et al., 2017; Geeraerts et al., 2017; Raimondi et al., 2018; Li et al., 2019; Mansur et al., 2019; Peruzy et al., 2019), these bacteria were inoculated on irradiated MP samples, in mono- and in co-culture experiments.

However, the selection of dominant and non-dominant species in inoculation experiments could have been more interesting in order to better represent the natural contamination of MP, and thus to better model the impact of sub-dominant microbiota. Indeed, others taxa were also present in MP samples but in lesser abundance, even if they are considered as dominant taxa in several studies: *Photobacterium* spp. (Ast et al., 2007; Bjornsdottir-Butler et al., 2016; Moretro et al., 2016; Nieminen et al., 2016; Kuuliala et al., 2018; Fogarty et al., 2019; Jääskeläinen et al., 2019) and *Lactobacillus* spp. (especially *Lactobacillus algidus*) (Kato et al., 2000; Fadda et al., 2010; Doulgeraki et al., 2012; Dalcanton et al., 2013; Nieminen et al., 2015; Pothakos et al., 2015; Alvarez-Sieiro et al., 2016; Woraprayote et al., 2016; Stefanovic et al., 2017). According to this, they were not included in models of this study, as all others non-dominant microbiota. Moreover, *P. fluorescens* and *P. fragi* were used together in experiments. The objective of this study was to offer an exploratory approach to the proposed method by following the common genus formed by the two species mentioned. So, it would have been interesting to inoculate MP samples with both species in different batches, as behavior of these species is different according to the storage conditions.

The inputs of models were provided from culture-dependent and culture-independent analysis performed on inoculation experiments. The association of both techniques allows us to obtain estimate abundance during storage in co-culture experiments. Although we acknowledge that the plate count method is not able to assess all the microbial populations in presence, the combination of these two methods was previously validated by a qPCR approach (Cauchie et al., 2017). This approach was also used in others studies (Chaillou et al., 2015; Delhalle et al., 2016). Fougy et al. (2016) also showed that this conversion can be used to obtain an extrapolated estimation of the bacterial concentration, and may be used in food industries. But comparison of these results with counts on selective media would also be interesting to study in the future. Moreover, even if this method overestimates the bacterial concentration, it could be beneficial in a worst-case risk assumption for food industries (Crotta et al., 2016; Membré and Boué, 2018).

In this study, models show relatively good fitting indexes (*RrMSE* and *R*^2^). Good performances (*ASZ*) in the three-species interaction approach were also obtained, especially with the modified Jameson-effect model.

The growth parameters of the three specific spoilage micro-organisms were obtained for mono- and co-culture experiments by fittings primary and secondary models (Tables 4, 5). The food packaging shows the highest impact on bacterial growth rates (μ_*max*_), which in turn have the strongest influence on the shelf life of food products (Simpson and Carevic, 2004; Stoops et al., 2015; Guillard et al., 2016; Saraiva et al., 2016; Couvert et al., 2017). In accordance with Liu et al. (2006), *N*_0_ showed a little correlation with the microbial shelf life in mono- and co-culture experiments, indicated that the storage outcome of food seems to be not completely determined by the initial microbial counts. Moreover, no obvious correlation has been shown between *N*_*max*_ and shelf life in co-cultures experiments. This can be explained by the fact that meat shelf life is determined primarily by the metabolic patterns of the spoilage microbiota, rather than by total counts of bacteria (Liu et al., 2006). However, it can be observed that the parameters obtained in single culture were quite different from those in co-culture, especially for *Pseudomonas* spp. and *B. thermosphacta*. In FW, *B. thermosphacta* grew faster on mono-culture, but this behavior was not detected in co-culture. On the opposite, *Pseudomonas* spp. became the dominant bacteria in FW in the presence of the two others micro-organisms. These differences between mono- and co-culture inoculations have already been observed by Hibbing et al. (2010) and Quinto et al. (2018).

On the other hand, observations in co-culture experiments showed that the suppression of the two other bacteria occurred when the dominant one reached its *MCP*. This result reveals a potential Jameson effect between populations, rather than a prey-predator trend. According to these, differences between mono- and co-cultures experiments could maybe be explained by two hypotheses: (i) a non-specific interaction involving the Jameson effect, where growth inhibition is the result from a depletion in nutrient bioavailability and toxicity increase when the dominant bacteria reaches *N*_*MCP*_; and (ii) a specific interaction due to the modification of the food matrix where bacteria are growing (i.e., catabolism of carbon sources, the production of by products such as carbon dioxide and acids, …) (Bruce et al., 2017; Quinto et al., 2018; Correia Peres Costa et al., 2019; Kumariya et al., 2019). Nadell et al. (2016) have mentioned that *P. fluorescens* can produces extracellular matrix materials to give them an advantage over competitors. Quorum sensing could also be related to this inhibition by the dominant bacteria, by exchanging information to synchronize bacterial behavior in mixed-culture (Ng and Bassler, 2009; Dubey and Ben-Yehuda, 2011; Quinto et al., 2018).

The development of a three-spoilage species interaction model was then performed using two models: the modified Jameson-effect and the Lotka Volterra (Figures 6, 7). The modified Jameson-effect model showed slightly better fits than the Lotka Volterra equation, with 40–86% out of the observed counts falling into the *ASZ*, indicating a satisfactory model performance. It only concerns 14–48% for the prey-predator approach. These results can be explained by the fact that the dynamics of experimental and validation datasets seems to follow a Jameson behavior, because the minority bacteria decelerate when the majority one reaches the *MCP* (Cornu et al., 2011). Moreover, the modified Jameson-effect equation is considering growth parameters *(*μ_*max*_, *t*_*MCP*_, and *N*_0_) for modeling (Eq. 19). These parameters are obtained by primary and secondary fittings, and are relatively reliable in our study due to the numbers of samples analyzed. On the other hand, the Lotka Volterra model is based on complex interaction factors (Eq. 21) which are obtained by linear regression. Due to the high variability of interactions that can be simulated, particularly in three or more species models, these interaction factors must necessarily be as accurate as possible. In this study, interaction factors are included in highly variable intervals (Table 8), with some variations observed according to the temperature (Moller et al., 2013; Mejlholm and Dalgaard, 2015; Correia Peres Costa et al., 2019). More datasets are probably needed to obtained reliable factors. Also, the Lotka Volterra model could be modified for a more realistic approach by considering the effect of other influencing factors (e.g., environmental conditions such as several storage and packaging conditions, bacteriocin production, etc.) (Powell et al., 2004; Baka et al., 2014).

More inoculation experiments are so needed to develop better predictive models, especially for a three- or more-spoilage species interaction approach. And also, to better understand the dynamics of spoilage bacteria toward each other and in the presence of natural microbiota. As mentioned by Quinto et al. (2018): “it is well known that a spoilage microorganism can either stimulate, inhibit, or have no effect on the growth of the pathogenic species.” So, it could be interesting to study interactions between spoilage microorganisms, with production of metabolites or other substances as interaction factors. It would also be interesting to investigate co-culture experiments with two species. Moreover, metabolites production by each of the inoculated bacteria, as inputs interacting models, will be studied in another scientific publication.

Finally, naturally contaminated samples were used to validate the developed models by considering the effect of the food microbiota. Differences with co-culture experiments were obtained: a predominance of *Photobacterium* spp. (>90% of reads) was observed in MAP (Supplementary Figure S5). It could be interesting to take also into account this bacterium for modeling interactions. The addition of this bacterium could possibly improve the reliability of predictions, particularly for the Lotka Volterra model. Moreover, *Photobacterium* spp. is not well recovered on PCA at 22°C (Dalgaard et al., 1997; Hilgarth et al., 2018). According to this, improving cultivation methods for this bacterium is important to obtain more reliable results. Further studies are so needed to develop more realistic interacting predictive models, especially in a three- or more-spoilage species interaction approach, and to develop new food preservation process.

## Conclusion

New omics technologies, such as metagenetics and metabolomics, are important to characterize and to follow the dynamics of bacterial microbiota and metabolites in complex food matrices. New generations of predictive models will probably need to be developed, by considering the results provided by these techniques. These models will provide a better understanding of the interactions between microorganisms and food, and micro-organisms between them.

## Data Availability Statement

All biosample raw reads were deposited at the National Center for Biotechnology Information (NCBI) and are available under de BioProject ID PRJNA590608.

## Author Contributions

EC did the experiments, interpreted the results, and wrote the manuscript. LD and GB performed the experiments, supervised analyses, and revisited the manuscript. BT, PF, FF, and GD were involved in the design of the study and provide help for interpretation of the results. AT and SB participated to the experiments. NK participated to the interpretation of the results and revisited of the manuscript. All authors read and approved the final manuscript.

## Conflict of Interest

SB and PF was employed by Quality Partner sa. The remaining authors declare that the research was conducted in the absence of any commercial or financial relationships that could be construed as a potential conflict of interest.

## Footnotes

1 http://microbiomeutil.sourceforge.net/#A_CS

2 http://www.arb-silva.de/
