# Supplementary material for: Modeling the Growth and Interaction Between Brochothrix thermosphacta, Pseudomonas spp., and Leuconostoc gelidum in Minced Pork Samples
Source: Front Microbiol. 2020 Apr 9;11:639. doi: 10.3389/fmicb.2020.00639 (PMC7160237; doi:10.3389/fmicb.2020.00639)
Supplement: Supplementary file 1 [file Data_Sheet_1.docx]

Supplementary Material

## Supplementary Figures

## Supplementary Figure 1. Inoculation experiments performed in food samples.

**
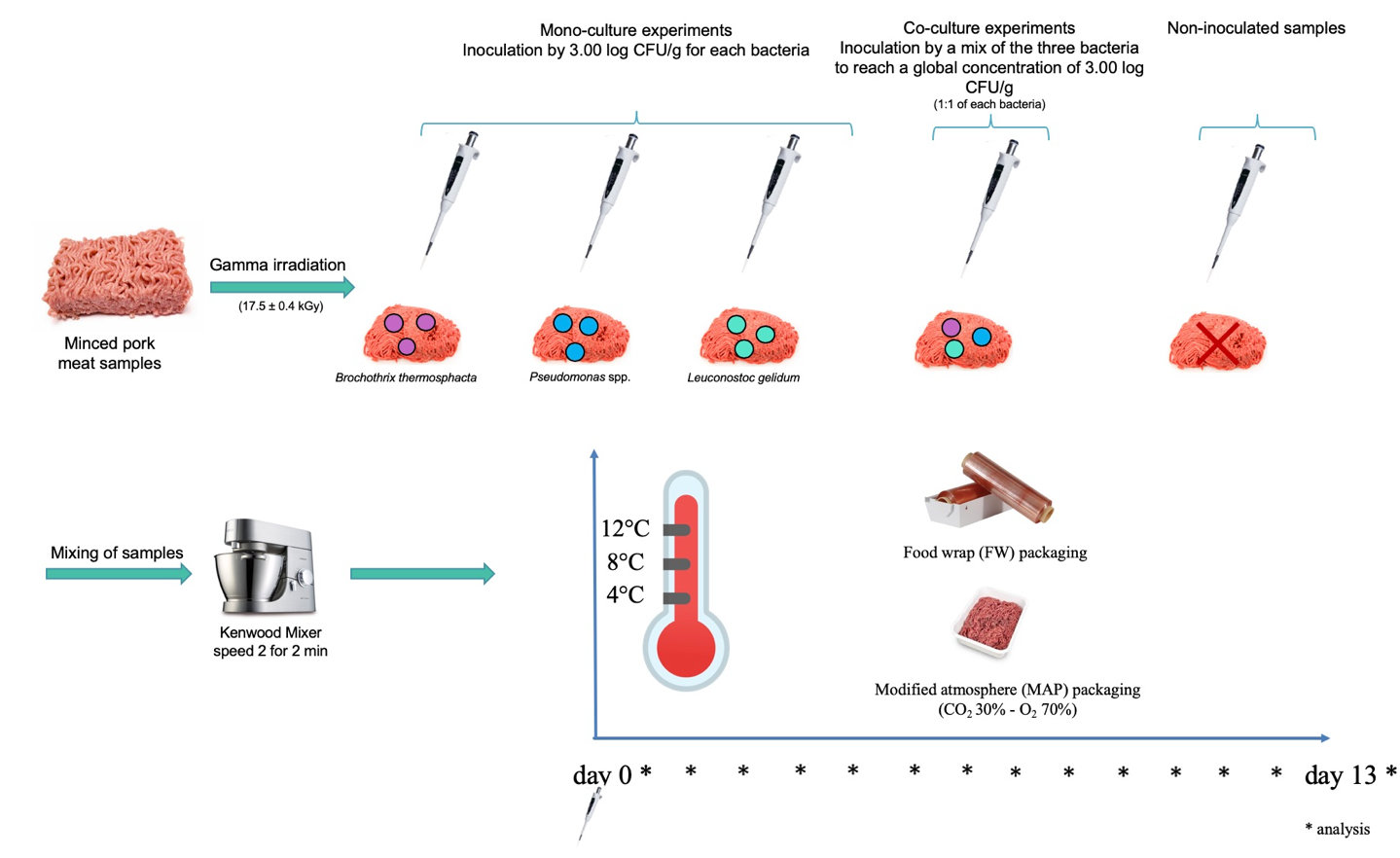
**

## Supplementary Figure 2. Cumulated histograms of the relative abundance (%) of taxa and the dynamics of the bacterial community identified by metagenetics at genus levels in inoculated minced pork meat samples (mono-culture experiments), at day 0 and 13, at 4°C in modified atmosphere (MAP) and food wrap (FW) packaging: (A) for *B. thermosphacta*, (B) for *Pseudomonas* spp., and (C) for *Ln. gelidum*. At genus levels, the taxa representing <1% in relative abundance were merged in the category of "Others".


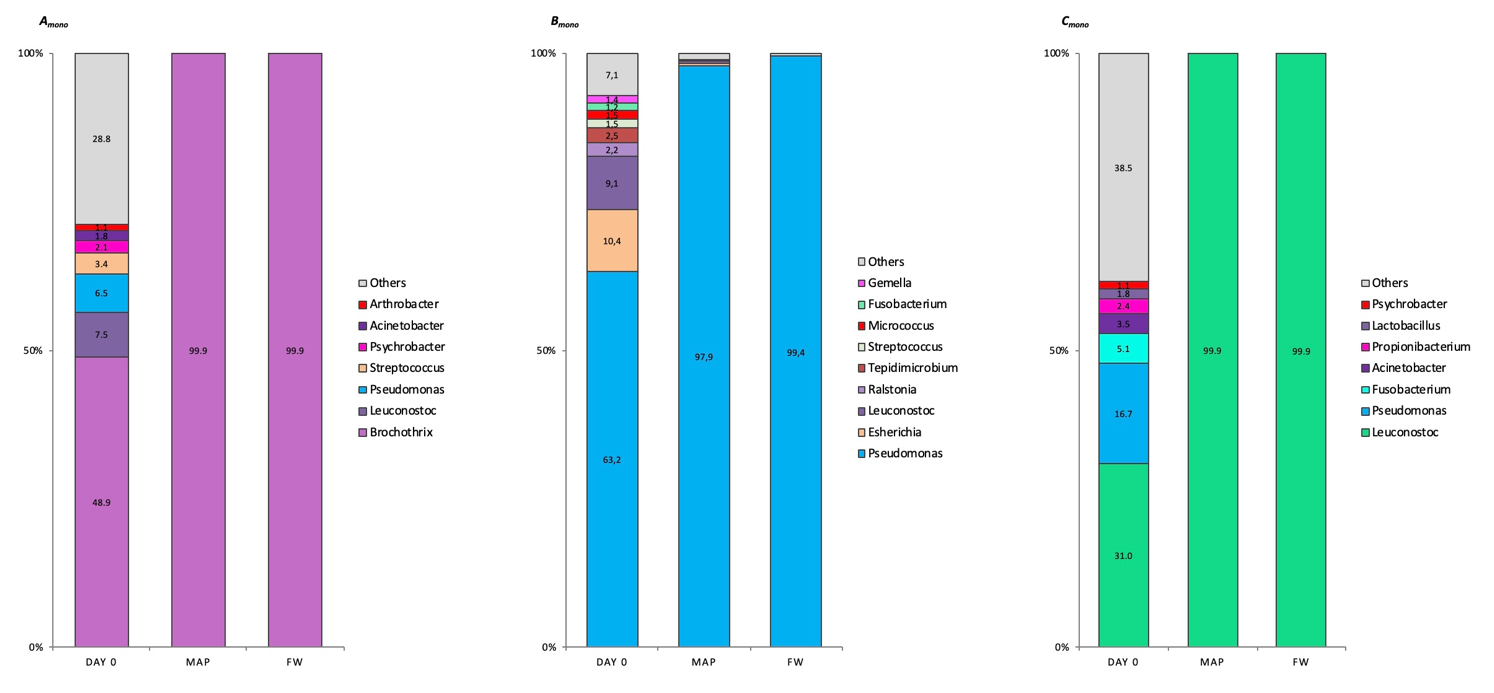


**Supplementary Figure 3.** Comparison of pH values for control samples with mono- and co-culture experiments, at day 13 for all packaging conditions, FW (food wrap packaging), MAP (modified atmosphere packaging), * significant statistical difference (p-value < 0.05).


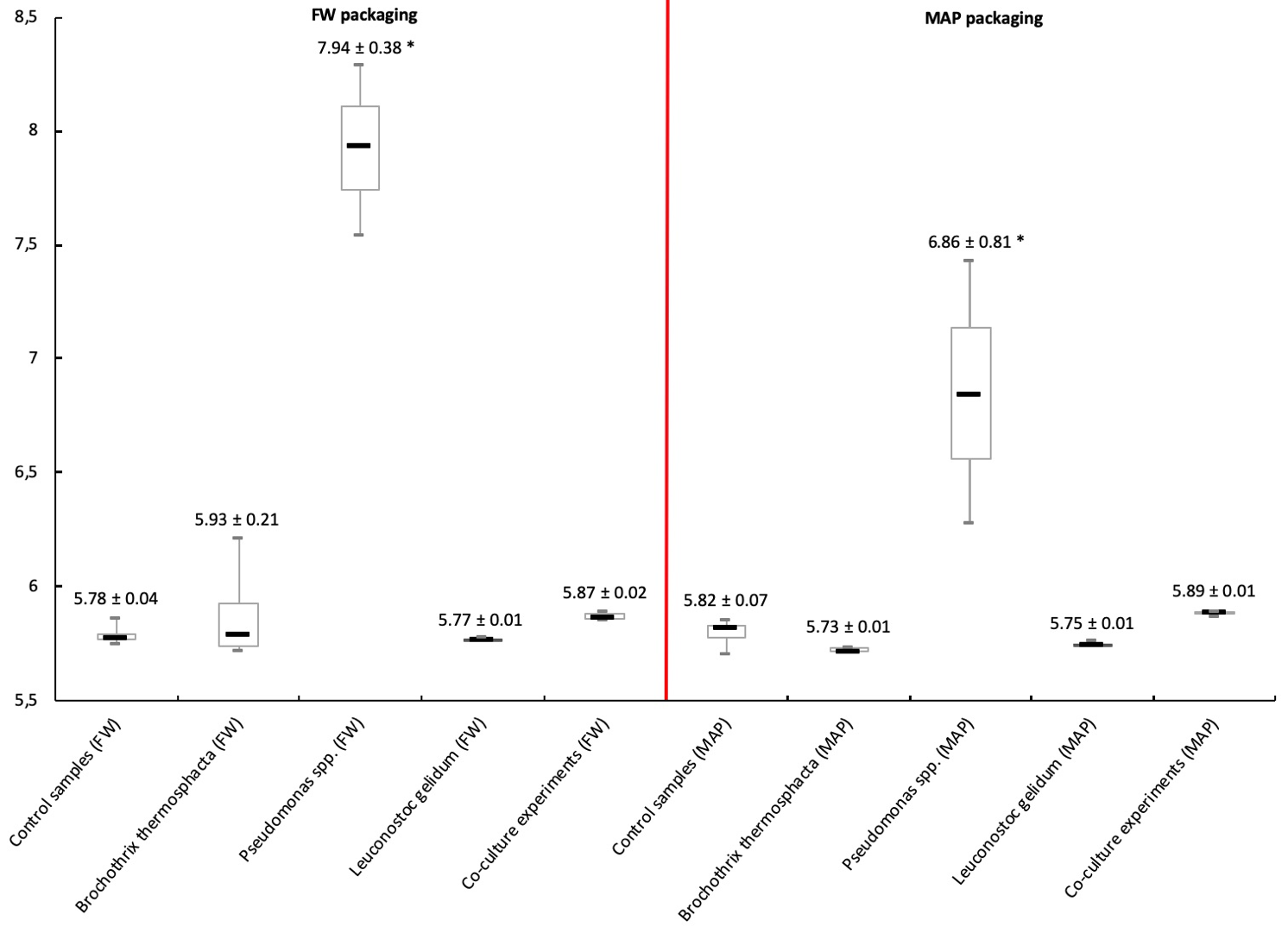


**Supplementary Figure 4.** Comparison of carbon dioxygen measurements for control samples with mono- and co-culture experiments, at day 13 for modified atmosphere packaging conditions, * significant statistical difference (p-value < 0.05).


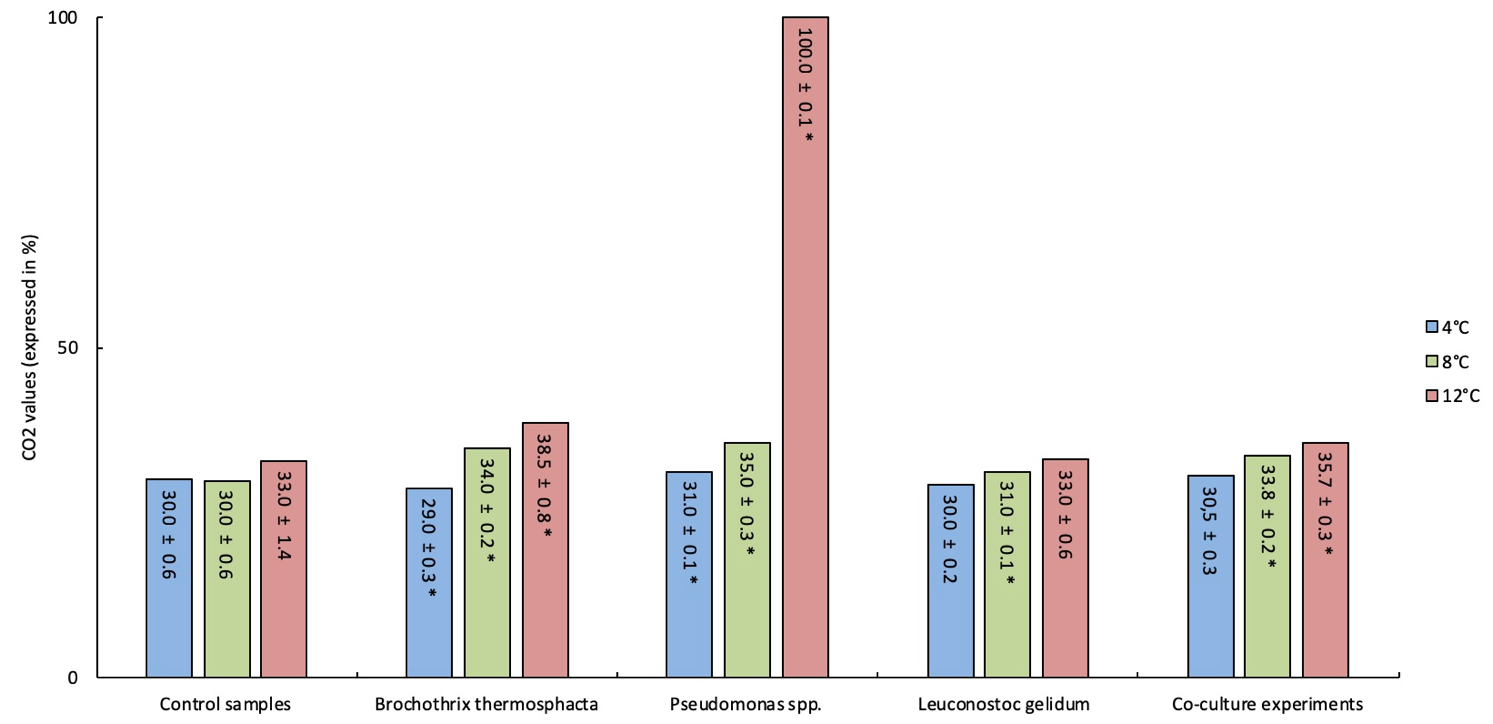


**Supplementary Figure 5.** Cumulated histograms of the relative abundance (%) of taxa and the dynamics of the bacterial community identified by metagenetics at species levels in validation dataset during storage in food wrap (**A**, at 4°C; **B**, at 8°C; **C**, at 12°C) At species levels, the taxa representing <1% in relative abundance were merged in the category of "Others". The solid represents the plate counts (means and standard deviation of the three replicates).


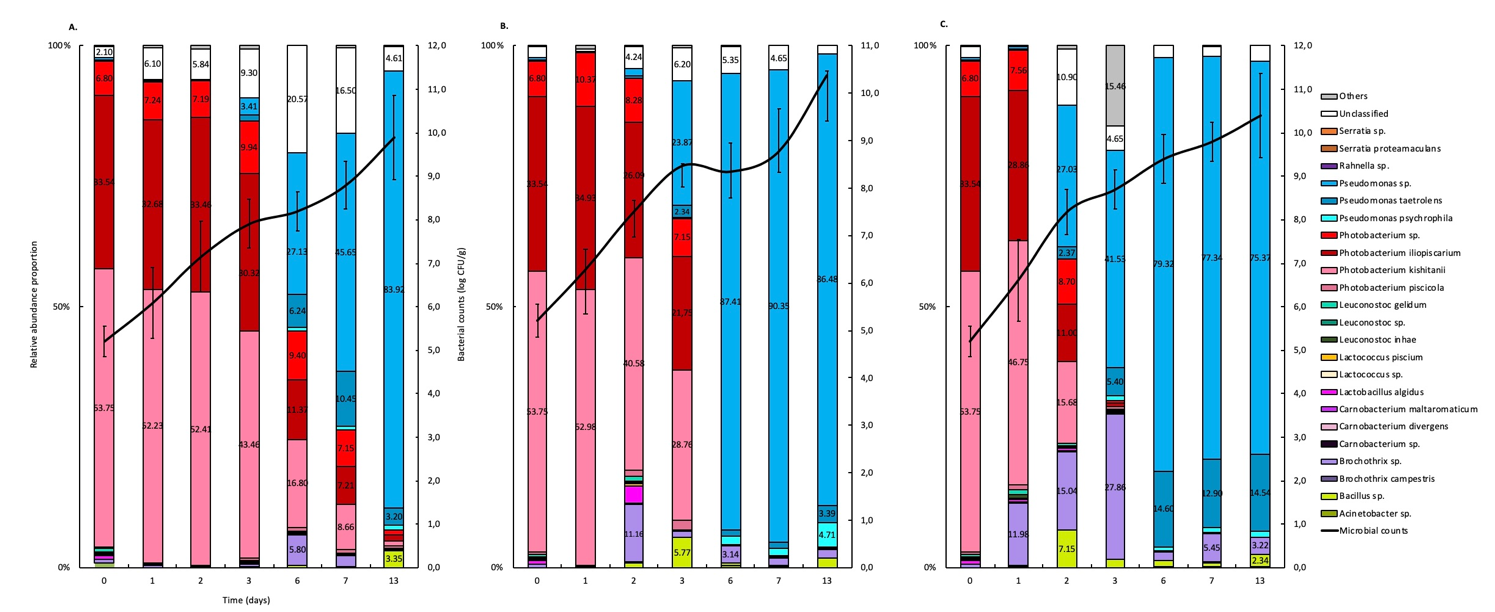


**Supplementary Figure 6.** Cumulated histograms of the relative abundance (%) of taxa and the dynamics of the bacterial community identified by metagenetics at species levels in validation dataset during storage in modified atmosphere packaging (**D**, at 4°C; **E**, at 8°C; **F**, at 12°C). At species levels, the taxa representing <1% in relative abundance were merged in the category of "Others". The solid represents the plate counts (means and standard deviation of the three replicates).


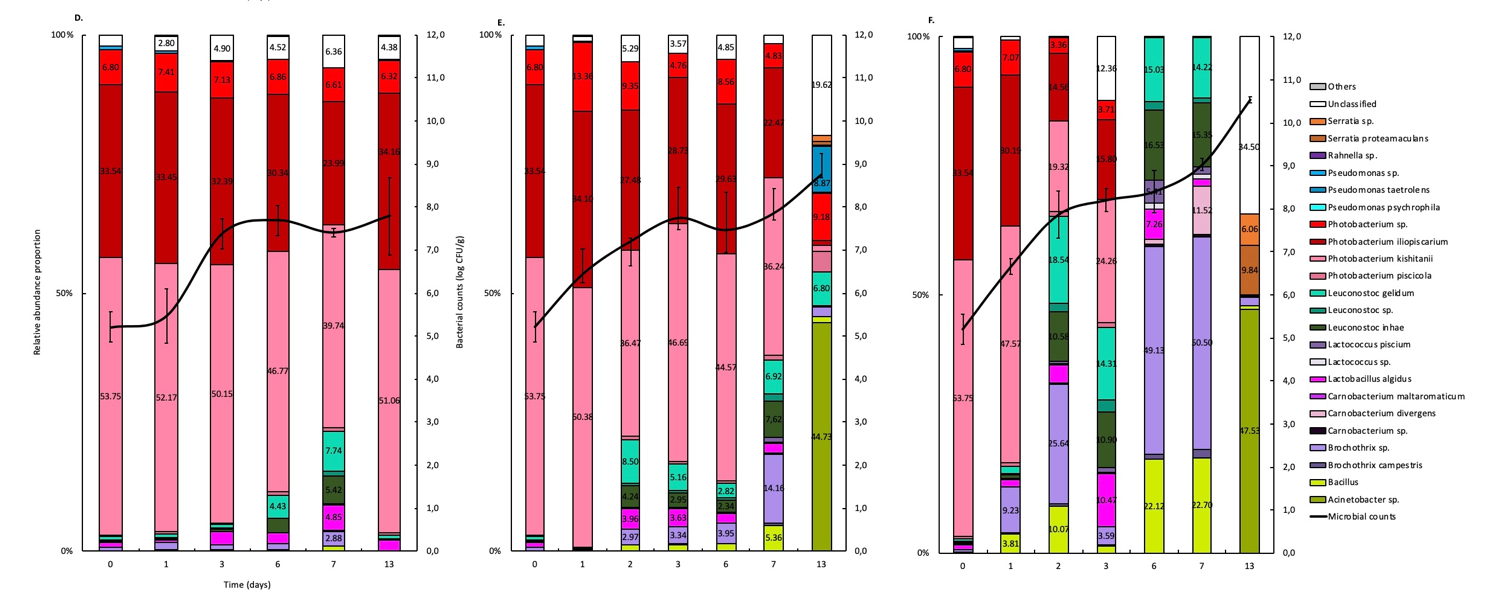


1. **Supplementary Tables**

**Supplementary Table 1.** Goodness-of-fit indexes for the two-species modified Jameson-effect model on mono-culture experiment data, by using Baranyi, Buchanan and without-lag functions.

| **Two-species Model** | ***RrMSE*** | | |
| --- | --- | --- | --- |
|  | **Baranyi** | **Buchanan** | **without-lag** |
| *A_mono_*-*G_mono_* | 0.3528 | - ^a^ | 0.5787 |
| *A_mono_*-*M_mono_* | - ^a^ | - ^a^ | 0.6879 |
| *G_mono_*-*M_mono_* | - ^a^ | - ^a^ | 0.3640 |
| *B_mono_*-H_mono_ | 0.3870 | - ^a^ | 0.8679 |
| *B_mono_*-*N_mono_* | 0.1284 | - ^a^ | 0.7059 |
| *H_mono_*-*N_mono_* | 0.1821 | 0.1830 | 0.3422 |
| *C_mono_*-*I_mono_* | 0.2486 | - ^a^ | 0.7820 |
| *C_mono_*-*O_mono_* | 0.5000 | - ^a^ | 0.7681 |
| *I_mono_*-*O_mono_* | - ^a^ | - ^a^ | 0.4453 |
| *D_mono_*-*J_mono_* | - ^a^ | - ^a^ | 0.4690 |
| *D_mono_*-*P_mono_* | - ^a^ | - ^a^ | 0.4743 |
| *J_mono_*-*P_mono_* | - ^a^ | - ^a^ | 0.2591 |
| *Q_mono_*-*E_mono_* | 0.1668 | - ^a^ | 0.3046 |
| *Q_mono_*-*K_mono_* | 0.6390 | - ^a^ | 0.6867 |
| *E_mono_*-*K_mono_* | 0.6390 | - ^a^ | 0.8276 |
| *F_mono_*-*L_mono_* | 0.2665 | - ^a^ | 0.6745 |
| *F_mono_*-*R_mono_* | 0.3011 | - ^a^ | 0.3502 |
| *L_mono_*-*R_mono_* | - ^a^ | - ^a^ | 1.0511 |

*RrMSE*, the root mean square error of the residuals mean sum of square; -^a^, no bacterial fitting obtained.

**Supplementary Table 2.** Distribution of metagenetics reads percentages at genus level for co-culture experiments.

| **Genus** | **Days** | | | | | | | | |
| --- | --- | --- | --- | --- | --- | --- | --- | --- | --- |
|  | **0** | **1** | **2** | **3** | **4** | **5** | **6** | **7** | **13** |
| FW | | | | | | | | | |
| 4°C |  |  |  |  |  |  |  |  |  |
| *Brochothrix* | 11.80 | 9.10 | 0.75 | - ^a^ | - ^a^ | - ^*^ | - ^*^ | - ^*^ | 0.52 |
| *Lactobacillus* | 10.96 | - ^*^ | - ^*^ | - ^a^ | - ^a^ | - ^*^ | - ^*^ | - ^*^ | 0.10 |
| *Leuconostoc* | 23.29 | 5.30 | 2.35 | - ^a^ | - ^a^ | 0.10 | 0.10 | 0.05 | 0.07 |
| *Photobacterium* | 21.92 | - ^*^ | - ^*^ | - ^a^ | - ^a^ | - ^*^ | - ^*^ | - ^*^ | - ^*^ |
| *Pseudomonas* | 27.40 | 64.40 | 93.44 | - ^a^ | - ^a^ | 99.80 | 99.80 | 98.51 | 98.25 |
| *Rahnella* | 4.11 | - ^*^ | - ^*^ | - ^a^ | - ^a^ | - ^*^ | - ^*^ | - ^*^ | - ^*^ |
| Others | 0.53 | 21.20 | 3.45 | - ^a^ | - ^a^ | 0.10 | 0.10 | 1.42 | 1.06 |
| 8°C |  |  |  |  |  |  |  |  |  |
| *Brochothrix* | 11.80 | 2.90 | - ^*^ | - ^a^ | - ^*^ | - ^*^ | 1.00 | 0.43 | 0.52 |
| *Lactobacillus* | 10.96 | - ^*^ | - ^*^ | - ^a^ | - ^*^ | - ^*^ | - ^*^ | - ^*^ | 0.10 |
| *Leuconostoc* | 23.29 | 4.80 | 0.07 | - ^a^ | - ^*^ | 0.20 | 0.30 | 0.08 | 0.07 |
| *Photobacterium* | 21.92 | - ^*^ | - ^*^ | - ^a^ | - ^*^ | - ^*^ | - ^*^ | - ^*^ | - ^*^ |
| *Pseudomonas* | 27.40 | 83.30 | 98.80 | - ^a^ | 97.10 | 96.50 | 94.50 | 97.85 | 98.25 |
| *Rahnella* | 4.11 | - ^*^ | - ^*^ | - ^a^ | - ^*^ | - ^*^ | - ^*^ | - ^*^ | - ^*^ |
| Others | 0.53 | 9.00 | 1.12 | - ^a^ | 2.90 | 3.30 | 4.20 | 1.65 | 1.06 |
| 12°C |  |  |  |  |  |  |  |  |  |
| *Brochothrix* | 11.80 | 0.40 | - ^*^ | - ^a^ | 1.40 | 1.90 | 0.60 | 0.20 | 0.10 |
| *Lactobacillus* | 10.96 | - ^*^ | - ^*^ | - ^a^ | - ^*^ | - ^*^ | - ^*^ | 0.03 | - ^*^ |
| *Leuconostoc* | 23.29 | 2.20 | 0.03 | - ^a^ | 0.20 | 0.70 | 0.10 | 0.07 | 0.07 |
| *Photobacterium* | 21.92 | - ^*^ | - ^*^ | - ^a^ | - ^*^ | - ^*^ | - ^*^ | - ^*^ | 0.07 |
| *Pseudomonas* | 27.40 | 93.90 | 99.41 | - ^a^ | 93.60 | 91.70 | 95.20 | 96.91 | 92.06 |
| *Rahnella* | 4.11 | - ^*^ | - ^*^ | - ^a^ | - ^*^ | - ^*^ | - ^*^ | - ^*^ | - ^*^ |
| Others | 0.53 | 3.50 | 0.55 | - ^a^ | 4.80 | 5.70 | 4.10 | 2.79 | 7.68 |
| MAP | | | | | | | | | |
| 4°C |  |  |  |  |  |  |  |  |  |
| *Brochothrix* | 11.80 | 10.10 | - ^a^ | 2.09 | - ^a^ | - ^a^ | 0.10 | - ^*^ | - ^*^ |
| *Lactobacillus* | 10.96 | - ^*^ | - ^a^ | 0.00 | - ^a^ | - ^a^ | - ^*^ | 0.03 | - ^*^ |
| *Leuconostoc* | 23.29 | 23.90 | - ^a^ | 46.23 | - ^a^ | - ^a^ | 94.20 | 99.74 | 99.92 |
| *Photobacterium* | 21.92 | - ^*^ | - ^a^ | 0.00 | - ^a^ | - ^a^ | - ^*^ | - ^*^ | - ^*^ |
| *Pseudomonas* | 27.40 | 29.40 | - ^a^ | 39.0 | - ^a^ | - ^a^ | 0.20 | 0.23 | 0.03 |
| *Rahnella* | 4.11 | - ^*^ | - ^a^ | 0.00 | - ^a^ | - ^a^ | - ^*^ | - ^*^ | - ^*^ |
| Others | 0.53 | 36.60 | - ^a^ | 12.67 | - ^a^ | - ^a^ | 5.50 | - ^*^ | 0.05 |
| 8°C |  |  |  |  |  |  |  |  |  |
| *Brochothrix* | 11.80 | 8.10 | 2.80 | 0.50 | - ^a^ | - ^a^ | - ^*^ | - ^*^ | 0.03 |
| *Lactobacillus* | 10.96 | - ^*^ | - ^*^ | - ^*^ | - ^a^ | - ^a^ | - ^*^ | - ^*^ | - ^*^ |
| *Leuconostoc* | 23.29 | 39.10 | 65.0 | 90.89 | - ^a^ | - ^a^ | 95.40 | 99.94 | 99.92 |
| *Photobacterium* | 21.92 | - ^*^ | - ^*^ | - ^*^ | - ^a^ | - ^a^ | - ^*^ | 0.03 | - ^*^ |
| *Pseudomonas* | 27.40 | 30.80 | 24.20 | 7.95 | - ^a^ | - ^a^ | - ^*^ | 0.03 | 0.03 |
| *Rahnella* | 4.11 | - ^*^ | - ^*^ | - ^*^ | - ^a^ | - ^a^ | - ^*^ | - ^*^ | - ^*^ |
| Others | 0.53 | 22.00 | 8.00 | 0.76 | - ^a^ | - ^a^ | 4.60 | - ^*^ | 0.03 |
| 12°C |  |  |  |  |  |  |  |  |  |
| *Brochothrix* | 11.80 | 3.90 | 1.00 | 0 | - ^a^ | - ^a^ | 0.10 | 0.08 | 0.21 |
| *Lactobacillus* | 10.96 | - ^*^ | - ^*^ | - ^*^ | - ^a^ | - ^a^ | - ^*^ | 0.05 | 0.03 |
| *Leuconostoc* | 23.29 | 52.40 | 86.10 | 99.41 | - ^a^ | - ^a^ | 95.50 | 99.63 | 99.61 |
| *Photobacterium* | 21.92 | - ^*^ | - ^*^ | - ^*^ | - ^a^ | - ^a^ | - ^*^ | - ^*^ | - ^*^ |
| *Pseudomonas* | 27.40 | 34.40 | 10.20 | 0.20 | - ^a^ | - ^a^ | - ^*^ | 0.10 | 0.03 |
| *Rahnella* | 4.11 | - ^*^ | - ^*^ | - ^*^ | - ^a^ | - ^a^ | - ^*^ | - ^*^ | - ^*^ |
| Others | 0.53 | 9.30 | 2.70 | 0.39 | - ^a^ | - ^a^ | 4.40 | 0.10 | 0.13 |

At genus levels, the taxa representing <1% in relative abundance were merged in the category of “Others”, - ^*^ data under the detection limit, - ^a^ no analysis performed this day.

**Supplementary Table 3.** Goodness-of-fit indexes used in primary models for fitting the experimental data in mono- and co-culture experiments.

| **Models** | ***RrMSE*** | ***R^2^*** |
| --- | --- | --- |
| *A_mono_* | 0.0070 | 0.9996 |
| *B_mono_* | 0.0053 | 0.9997 |
| *C_mono_* | 0.0079 | 0.9994 |
| *D_mono_* | 0.0252 | 0.9943 |
| *E_mono_* | 0.0252 | 0.9943 |
| *F_mono_* | 0.0242 | 0.9947 |
| *G_mono_* | 0.0348 | 0.9891 |
| *H_mono_* | 0.0348 | 0.9891 |
| *I_mono_* | 0.0348 | 0.9891 |
| *J_mono_* | 0.0116 | 0.9988 |
| *K_mono_* | 0.0116 | 0.9988 |
| *L_mono_* | 0.0116 | 0.9988 |
| *M_mono_* | 0.0446 | 0.9821 |
| *N_mono_* | 0.0506 | 0.9770 |
| *O_mono_* | 0.0446 | 0.9821 |
| *P_mono_* | 0.0533 | 0.9744 |
| *Q_mono_* | 0.0513 | 0.9763 |
| *R_mono_* | 0.0533 | 0.9744 |
| A_co(A)_ | 0.0074 | 0.9995 |
| A_co(B)_ | 0.1045 | 0.9018 |
| A_co(C)_ | 0.0172 | 0.9974 |
| B_co(A)_ | 0.0394 | 0.9860 |
| B_co(B)_ | 0.2290 | 0.4280 |
| B_co(C)_ | 0.0428 | 0.9835 |
| C_co(A)_ | 0.1140 | 0.8830 |
| C_co(B)_ | 0.2290 | 0.5280 |
| C_co(C)_ | 0.0097 | 0.9992 |
| D_co(A)_ | 0.0041 | 0.9999 |
| D_co(B)_ | 0.1226 | 0.8647 |
| D_co(C)_ | 0.0722 | 0.9531 |
| E_co(A)_ | 0.0249 | 0.9944 |
| E_co(B)_ | 0.0811 | 0.9408 |
| E_co(C)_ | 0.0924 | 0.9231 |
| F_co(A)_ | 0.0265 | 0.9937 |
| F_co(B)_ | 0.0414 | 0.9846 |
| F_co(C)_ | 0.0585 | 0.9692 |

*RrMSE*, the root mean square error of the residuals (standard deviation of the residuals); *R^2^*, the coefficient of multiple determination (the fraction of the square of the deviations of the observed values about their mean explained by the equation fitted to the experimental data).

**Supplementary Table 4.** Goodness-of-fit indexes used in secondary models for fitting the experimental data in mono- and co-culture experiments.

| **Mono-culture experiments** | | ***GoF*** | ***R^2^*** |
| --- | --- | --- | --- |
| FW | *B. thermosphacta* | 0.0183 | 0.9993 |
| FW | *Pseudomonas* spp. | 0.0231 | 0.9989 |
| FW | *L. gelidum* | 0.0479 | 0.9954 |
| MAP | *B. thermosphacta* | 0.0012 | 1.0000 |
| MAP | *Pseudomonas* spp. | 0.0127 | 0.9997 |
| MAP | *L. gelidum* | 0.0041 | 0.9989 |
|  |  |  |  |
| **Co-culture experiments** | |  |  |
| FW | *B. thermosphacta* | 0.0098 | 0.9998 |
| FW | *Pseudomonas* spp. | 0.0097 | 0.9998 |
| FW | *L. gelidum* | 0.0020 | 1.0000 |
| MAP | *B. thermosphacta* | 0.0068 | 0.9999 |
| MAP | *Pseudomonas* spp. | 0.1075 | 0.9769 |
| MAP | *L. gelidum* | 0.0194 | 0.9993 |

*GoF*, the goodness of fit (root meat square error of the model, analogous to the accuracy factor*)*; *R^2^* , the coefficient of multiple determination (the fraction of the square of the deviations of the observed values about their mean explained by the equation fitted to the experimental data).

**Supplementary Table 5.** Distribution of metagenetic reads percentages at species level for validation dataset, during storage of minced pork meat samples in food wrap packaging.

| **Temp.** | **Days** | ***Acinetobacter* sp.** | ***Bacillus* sp.** | ***Brochothrix campestris*** | ***Brochothrix* sp.** | ***Carnobacterium* sp.** | ***Carnobacterium divergens*** | ***Carnobacterium maltaromaticum*** | ***Lactobacillus algidus*** | ***Lactococcus* sp.** | ***Lactococcus piscium*** | ***Leuconostoc inhae*** | ***Leuconotos* sp.** | ***Leuconostoc gelidum*** | ***Photobacterium kishitanii*** | ***Photobacterium illiopiscarium*** | ***Photobacterium* sp.** | ***Photobacterium piscicola*** | ***Pseudomonas psychrophila*** | ***Pseudomonas taetrolens*** | ***Pseudomonas* sp.** | ***Rahnella* sp.** | ***Serratia proteamaculans*** | ***Serratia* sp.** | **Unclassified** | **Others** |
| --- | --- | --- | --- | --- | --- | --- | --- | --- | --- | --- | --- | --- | --- | --- | --- | --- | --- | --- | --- | --- | --- | --- | --- | --- | --- | --- |
| 4°C | 0 | - * | - * | - * | 0.56 | - * | 0.03 | - * | 0.86 | 0.18 | 0.12 | 0.21 | 0.12 | 0.59 | 0.35 | 53.75 | 33.54 | 6.80 | 0.03 | 0.06 | 0.56 | - * | - * | - * | 2.10 | 0.15 |
|  | 1 | - * | - * | - * | 0.45 | - * | - * | 0.03 | 0.05 | 0.03 | 0.08 | - * | - * | 0.19 | 0.19 | 52.23 | 32.68 | 7.24 | - * | 0.11 | 0.24 | - * | - * | - * | 6.10 | 0.39 |
|  | 2 | - * | - * | - * | 0.17 | - * | 0.03 | - * | - * | - * | - * | - * | - * | 0.03 | 0.11 | 52.41 | 33.46 | 7.19 | - * | - * | 0.14 | - * | - * | - * | 5.84 | 0.63 |
|  | 3 | - * | 0.09 | - * | 0.69 | 0.03 | 0.03 | - * | 0.21 | 0.03 | 0.03 | 0.03 | 0.03 | 0.15 | 0.51 | 43.46 | 30.32 | 9.94 | 0.15 | 1.02 | 3.41 | - * | - * | - * | 9.30 | 0.58 |
|  | 6 | 0.03 | 0.52 | - * | 5.80 | - * | - * | - * | 0.17 | 0.06 | 0.09 | 0.09 | - * | 0.29 | 0.73 | 16.80 | 11.37 | 9.40 | 0.73 | 6.24 | 27.13 | - * | - * | - * | 20.57 | 0.00 |
|  | 7 | - * | 0.25 | - * | 2.13 | 0.03 | - * | - * | 0.09 | 0.06 | 0.09 | - * | - * | 0.09 | 0.68 | 8.66 | 7.21 | 7.15 | 0.62 | 10.45 | 45.65 | - * | - * | - * | 16.50 | 0.33 |
|  | 13 | - * | 3.35 | - * | 0.09 | - * | - * | - * | 0.15 | - * | - * | 0.03 | - * | - * | 0.53 | 1.06 | 1.12 | 0.88 | 0.94 | 3.20 | 83.92 | - * | - * | - * | 4.61 | 0.15 |
|  |  |  |  |  |  |  |  |  |  |  |  |  |  |  |  |  |  |  |  |  |  |  |  |  |  |  |
| 8°C | 0 | - * | - * | - * | 0.56 | - * | 0.03 | - * | 0.86 | 0.18 | 0.12 | 0.21 | 0.12 | 0.59 | 0.35 | 53.75 | 33.54 | 6.80 | 0.03 | 0.06 | 0.56 | - * | - * | - * | 2.10 | 0.15 |
|  | 1 | - * | 0.06 | - * | 0.17 | - * | - * | - * | - * | - * | - * | - * | - * | 0.06 | 0.14 | 52.98 | 34.93 | 10.37 | - * | 0.03 | 0.22 | - * | - * | - * | 0.47 | 0.58 |
|  | 2 | - * | 0.90 | 0.13 | 11.16 | 0.10 | 0.07 | - * | 3.27 | 0.07 | 0.33 | 0.40 | 0.23 | 0.80 | 1.30 | 40.58 | 26.09 | 8.28 | 0.03 | 0.43 | 1.50 | - * | - * | - * | 4.24 | 0.07 |
|  | 3 | - * | 5.77 | - * | 1.17 | - * | - * | - * | 0.07 | - * | - * | 0.15 | - * | - * | 2.04 | 28.76 | 21.75 | 7.15 | 0.29 | 2.34 | 23.87 | - * | - * | - * | 6.20 | 0.44 |
|  | 6 | 0.37 | 0.55 | 0.05 | 3.14 | 0.02 | 0.05 | - * | - * | - * | 0.02 | 0.02 | - * | 0.02 | - * | 0.12 | 0.02 | 0.02 | 1.62 | 1.14 | 87.41 | - * | - * | - * | 5.35 | 0.05 |
|  | 7 | 0.09 | 0.42 | 0.02 | 1.39 | - * | 0.02 | - * | 0.02 | - * | - * | - * | - * | - * | - * | 0.14 | 0.12 | 0.02 | 1.53 | 1.20 | 90.35 | - * | - * | - * | 4.65 | 0.02 |
|  | 13 | 0.03 | 1.75 | - * | 1.78 | - * | - * | - * | - * | - * | - * | - * | - * | - * | 0.03 | 0.19 | 0.11 | 0.03 | 4.71 | 3.39 | 86.48 | - * | - * | - * | 1.51 | 0.00 |
|  |  |  |  |  |  |  |  |  |  |  |  |  |  |  |  |  |  |  |  |  |  |  |  |  |  |  |
| 12°C | 0 | - * | - * | - * | 0.56 | - * | 0.03 | - * | 0.86 | 0.18 | 0.12 | 0.21 | 0.12 | 0.59 | 0.35 | 53.75 | 33.54 | 6.80 | 0.03 | 0.06 | 0.56 | - * | - * | - * | 2.10 | 0.15 |
|  | 1 | - * | 0.32 | 0.10 | 11.98 | - * | 0.13 | - * | 0.64 | - * | 0.19 | 0.54 | 0.19 | 0.89 | 0.96 | 46.75 | 28.86 | 7.56 | - * | 0.26 | 0.41 | - * | - * | - * | 0.11 | 0.13 |
|  | 2 | - * | 7.15 | - * | 15.04 | - * | 0.14 | - * | 0.53 | - * | 0.11 | 0.21 | 0.14 | 0.42 | - * | 15.68 | 11.00 | 8.70 | - * | 2.37 | 27.03 | - * | - * | - * | 10.90 | 0.57 |
|  | 3 | - * | 1.50 | - * | 27.86 | 0.03 | - * | - * | 0.25 | - * | 0.37 | 0.09 | 0.06 | 0.12 | - * | 0.56 | 0.78 | 0.34 | 0.99 | 5.40 | 41.53 | - * | - * | - * | 4.65 | 15.46 |
|  | 6 | 0.24 | 1.16 | 0.06 | 1.66 | 0.03 | - * | 0.06 | - * | - * | - * | - * | - * | - * | - * | - * | - * | - * | 0.74 | 14.60 | 79.32 | - * | - * | - * | 2.14 | 0.00 |
|  | 7 | 0.12 | 0.84 | 0.09 | 5.45 | - * | 0.09 | 0.12 | 0.03 | - * | - * | 0.03 | 0.06 | - * | - * | - * | 0.03 | - * | 0.93 | 12.90 | 77.34 | - * | - * | - * | 1.89 | 0.09 |
|  | 13 | 0.19 | 2.34 | 0.06 | 3.22 | - * | - * | - * | - * | - * | - * | - * | - * | 0.03 | - * | - * | - * | - * | 1.20 | 14.54 | 75.37 | - * | 0.06 | - * | 2.94 | 0.00 |

At species levels, the taxa representing <1% in relative abundance were merged in the category of “Others”, Temp. (temperature, °C), - ^*^ data under the detection limit.

**Supplementary Table 6.** Distribution of metagenetic reads percentages at species level for validation dataset, during storage of minced pork meat samples in modified atmosphere packaging.

| **Temp.** | **Days** | ***Acinetobacter* sp.** | ***Bacillus* sp.** | ***Brochothrix campestris*** | ***Brochothrix* sp.** | ***Carnobacterium* sp.** | ***Carnobacterium divergens*** | ***Carnobacterium maltaromaticum*** | ***Lactobacillus algidus*** | ***Lactococcus* sp.** | ***Lactococcus piscium*** | ***Leuconostoc inhae*** | ***Leuconotos* sp.** | ***Leuconostoc gelidum*** | ***Photobacterium kishitanii*** | ***Photobacterium illiopiscarium*** | ***Photobacterium* sp.** | ***Photobacterium piscicola*** | ***Pseudomonas psychrophila*** | ***Pseudomonas taetrolens*** | ***Pseudomonas* sp.** | ***Rahnella* sp.** | ***Serratia proteamaculans*** | ***Serratia* sp.** | **Unclassified** | **Others** |
| --- | --- | --- | --- | --- | --- | --- | --- | --- | --- | --- | --- | --- | --- | --- | --- | --- | --- | --- | --- | --- | --- | --- | --- | --- | --- | --- |
| 4°C | 0 | - * | - * | - * | 0.56 | - * | 0.03 | - * | 0.86 | 0.18 | 0.12 | 0.21 | 0.12 | 0.59 | 0.35 | 53.75 | 33.54 | 6.80 | 0.03 | 0.06 | 0.56 | - * | - * | - * | 2.10 | 0.15 |
|  | 1 | - * | - * | - * | 1.23 | - * | - * | 0.14 | 0.36 | 0.06 | 0.03 | 0.22 | 0.14 | 0.78 | 0.42 | 52.17 | 33.45 | 7.41 | - * | 0.11 | 0.45 | - * | - * | - * | 2.80 | 0.24 |
|  | 2 | - ^a^ | - ^a^ | - ^a^ | - ^a^ | - ^a^ | - ^a^ | - ^a^ | - ^a^ | - ^a^ | - ^a^ | - ^a^ | - ^a^ | - ^a^ | - ^a^ | - ^a^ | - ^a^ | - ^a^ | - ^a^ | - ^a^ | - ^a^ | - ^a^ | - ^a^ | - ^a^ | - ^a^ | - ^a^ |
|  | 3 | - * | - * | - * | 0.92 | - * | - * | 0.03 | 2.49 | 0.03 | 0.09 | 0.31 | 0.31 | 0.74 | 0.22 | 50.15 | 32.39 | 7.13 | - * | 0.03 | 0.09 | - * | - * | - * | 4.90 | 0.17 |
|  | 6 | 0.03 | - * | - * | 0.97 | - * | - * | - * | 2.13 | 0.03 | - * | 2.76 | 0.21 | 4.43 | 0.67 | 46.77 | 30.34 | 6.86 | - * | - * | 0.06 | - * | - * | - * | 4.52 | 0.21 |
|  | 7 | - * | - * | - * | 2.88 | 0.16 | 0.06 | - * | 4.85 | 0.13 | 0.09 | 5.42 | 1.06 | 7.74 | 0.75 | 39.74 | 23.99 | 6.61 | - * | - * | 0.09 | - * | - * | - * | 6.36 | 0.06 |
|  | 13 | - * | 0.03 | - * | 0.12 | - * | - * | - * | 2.06 | - * | - * | 0.23 | - * | 0.70 | 0.46 | 51.06 | 34.16 | 6.32 | - * | - * | 0.23 | - * |  |  |  |  |
|  |  |  |  |  |  |  |  |  |  |  |  |  |  |  |  |  |  |  |  |  |  |  |  |  |  |  |
| 8°C | 0 | - * | - * | - * | 0.56 | - * | 0.03 | - * | 0.86 | 0.18 | 0.12 | 0.21 | 0.12 | 0.59 | 0.35 | 53.75 | 33.54 | 6.80 | 0.03 | 0.06 | 0.56 | - * | - * | - * | 2.10 | 0.15 |
|  | 1 | - * | - * | - * | 0.10 | - * | - * | - * | 0.12 | 0.02 | 0.15 | 0.10 | 0.07 | 0.12 | 0.07 | 50.38 | 34.10 | 13.36 | - * | - * | 0.05 | - * | - * | - * | 0.94 | 0.40 |
|  | 2 | - * | - * | 0.03 | 2.97 | 0.03 | - * | 0.06 | 3.96 | 0.06 | 0.21 | 4.24 | 0.51 | 8.50 | 0.76 | 36.47 | 27.48 | 9.35 | - * | 0.03 | 0.03 | - * | - * | - * | 5.29 | 0.00 |
|  | 3 | - * | - * | 0.06 | 3.34 | - * | - * | - * | 3.63 | - * | 0.23 | 2.95 | 0.34 | 5.26 | 0.51 | 46.69 | 28.73 | 4.76 | - * | - * | 0.06 | - * | - * | - * | 3.57 | 0.00 |
|  | 6 | - * | - * | - * | 3.95 | - * | - * | - * | 2.03 | 0.06 | 0.09 | 2.34 | 0.49 | 2.82 | 0.55 | 44.57 | 29.63 | 8.56 | - * | - * | 0.06 | - * | - * | - * | 4.85 | 0.00 |
|  | 7 | - * | 0.07 | 0.35 | 14.16 | 0.03 | - * | 0.07 | 2.09 | 0.21 | 0.83 | 7.62 | 1.39 | 6.92 | 0.83 | 36.24 | 22.47 | 4.83 | - * | 0.07 | - * | - * | - * | - * | 1.81 | 0.00 |
|  | 13 | 44.73 | 0.09 | - * | 2.07 | - * | - * | - * | - * | - * | - * | 0.09 | - * | 6.80 | 4.01 | 1.17 | 0.81 | 9.18 | 0.36 | 8.87 | 0.32 | - * | 0.59 | 1.31 | 19.62 | 0.00 |
|  |  |  |  |  |  |  |  |  |  |  |  |  |  |  |  |  |  |  |  |  |  |  |  |  |  |  |
| 12°C | 0 | - * | - * | - * | 0.56 | - * | 0.03 | - * | 0.86 | 0.18 | 0.12 | 0.21 | 0.12 | 0.59 | 0.35 | 53.75 | 33.54 | 6.80 | 0.03 | 0.06 | 0.56 | - * | - * | - * | 2.10 | 0.15 |
|  | 1 | - * | 0.03 | 0.16 | 9.23 | - * | - * | - * | 1.65 | - * | 0.16 | 0.82 | 0.19 | 1.46 | 0.73 | 47.57 | 30.19 | 7.07 | - * | 0.06 | 0.13 | - * | - * | - * | 0.54 | 0.00 |
|  | 2 | - * | - * | 0.39 | 25.64 | 0.06 | 0.03 | - * | 3.80 | 0.33 | 0.56 | 10.58 | 1.69 | 18.54 | 0.98 | 19.32 | 14.56 | 3.36 | - * | 0.03 | 0.09 | - * | - * | - * | - * | 0.03 |
|  | 3 | - * | - * | 0.06 | 3.59 | 0.03 | 0.03 | - * | 10.47 | 0.30 | 0.79 | 10.90 | 2.37 | 14.31 | 0.88 | 24.26 | 15.80 | 3.71 | - * | - * | 0.09 | - * | - * | - * | 12.36 | 0.03 |
|  | 6 | - * | - * | 1.19 | 49.13 | 0.60 | 1.06 | - * | 7.26 | 1.36 | 5.41 | 16.53 | 2.09 | 15.03 | 0.20 | 0.08 | 0.03 | 0.03 | - * | - * | - * | - * | - * | - * | - * | 0.00 |
|  | 7 | - * | - * | 2.04 | 50.50 | 0.47 | 11.52 | - * | 1.74 | 0.97 | 1.80 | 15.35 | 1.20 | 14.22 | - * | 0.07 | 0.07 | 0.03 | - * | - * | 0.03 | - * | - * | - * | - * | - * |
|  | 13 | 47.53 | - * | 0.04 | 1.53 | - * | - * | - * | - * | - * | - * | 0.27 | - *0.04 | - * | - * | - * | - * | - * | - * | 0.04 | 0.13 | - * | 9.84 | 6.06 | 34.50 | 0.00 |

At species levels, the taxa representing <1% in relative abundance were merged in the category of “Others”, Temp. (temperature, °C), - ^*^ data under the detection limit, - ^a^ no analysis performed this day.

**Supplementary Table 7.** Estimate bacterial counts calculated for validation dataset.

| Conditions/  Bacteria | Time (days) | | | | | | |
| --- | --- | --- | --- | --- | --- | --- | --- |
|  | **0** | **1** | **2** | **3** | **6** | **7** | **13** |
| FW 4°C |  |  |  | - ^a^ |  |  |  |
| *B.thermosphacta* | 2.96 [3.31-2.60] | 3.75 [4.10-2.94] | 4.37 [4.72-3.55] | 5.74 [6.09-5.18] | 6.96 [7.31-6.51] | 7.13 [7.48-6.58] | 6.84 [7.19-5.88] |
| *Pseudomonas* spp. | 3.02 [3.37-2.66] | 3.63 [3.98-2.83] | 4.29 [4.64-3.47] | 6.57 [6.92-6.01] | 7.73 [8.08-7.28] | 8.55 [8.90-8.00] | 9.84 [10.19-8.88] |
| *L. gelidum* | 3.06 [3.41-2.71] | - * | - * | 5.16 [5.51-4.60] | 5.66 [6.01-5.21] | - * | 5.68 [6.03-5.33] |
|  |  |  |  |  |  |  |  |
| FW 8°C |  |  |  |  |  |  |  |
| *B.thermosphacta* | 2.96 [3.31-2.60] | 3.51 [3.93-3.09] | 6.55 [6.80-6.31] | 6.54 [6.57-6.51] | 6.85 [7.44-6.26] | 6.92 [7.80-6.04] | 8.64 [8.71-8.57] |
| *Pseudomonas* spp. | 3.02 [3.37-2.66] | 3.68 [4.10-3.26] | 5.80 [6.04-5.56] | 7.90 [7.93-7.87] | 8.31 [8.90-7.72] | 8.74 [9.62-7.86] | 10.37 [10.44-10.30] |
| *L. gelidum* | 3.06 [3.41-2.71] | - * | 5.52 [5.76-5.28] | - * | 4.75 [5.34-4.16] | - * | - * |
|  |  |  |  |  |  |  |  |
| FW 12°C |  |  |  |  |  |  |  |
| *B.thermosphacta* | 2.96 [3.31-2.60] | 5.69 [6.63-4.75] | 7.36 [7.88-6.83] | 8.14 [8.59-7.69] | 7.62 [8.17-7.07] | 8.54 [8.99-8.09] | 8.91 [9.89-7.94] |
| *Pseudomonas* spp. | 3.02 [3.37-2.66] | 4.44 [5.38-3.50] | 7.65 [8.17-7.12] | 8.38 [8.83-7.93] | 9.38 [9.93-8.83] | 9.76 [10.21-9.31] | 10.36 [11.34-9.38] |
| *L. gelidum* | 3.06 [3.41-2.71] | 4.65 [5.59-3.71] | 5.93 [6.46-5.41] | 5.97 [6.42-5.52] | - * | 6.57 [7.02-6.12] | 6.59 [6.94-6.24] |
|  |  |  |  |  |  |  |  |
| MAP 4°C |  |  |  |  |  |  |  |
| *B.thermosphacta* | 2.96 [3.31-2.60] | 3.56 [3.91-2.94] | - * | 5.34 [5.69-4.99] | 5.67 [6.02-5.32] | 5.86 [6.21-5.76] | 4.85 [5.20-3.96] |
| *Pseudomonas* spp. | 3.02 [3.37-2.66] | 3.22 [3.57-2.60] | - * | 4.47 [4.82-4.11] | 4.47 [4.82-4.12] | 4.37 [4.72-4.27] | 5.15 [5.50-4.26] |
| *L. gelidum* | 3.06 [3.41-2.71] | 3.34 [3.79-2.81] | - * | 5.40 [5.75-5.04] | 6.35 [6.70-6.00] | 6.34 [6.69-6.24] | 5.63 [5.98-4.74] |
|  |  |  |  |  |  |  |  |
| MAP 8°C |  |  |  |  |  |  |  |
| *B.thermosphacta* | 2.96 [3.31-2.60] | 3.42 [4.02-2.82] | 5.66 [5.76-5.56] | 6.26 [6.99-5.52] | 6.05 6.94-5.16] | 6.99 [7.57-6.41] | 7.05 [7.56-6.54] |
| *Pseudomonas* spp. | 3.02 [3.37-2.66] | 3.12 [3.52-2.52] | 3.97 [4.07-3.87] | 4.49 [5.22-3.75] | 4.24 [5.13-3.35] | 4.68 [5.26-4.10] | 7.72 [8.23-7.21] |
| *L. gelidum* | 3.06 [3.41-2.71] | 3.72 [4.32-3.12] | 6.14 [6.24-6.04] | 6.47 [7.21-5.74] | 5.97 [6.86-5.08] | 6.76 [7.34-6.18] | 7.57 [8.08-7.06] |
|  |  |  |  |  |  |  |  |
| MAP 12°C |  |  |  |  |  |  |  |
| *B.thermosphacta* | 2.96 [3.31-2.60] | 5.62 [5.80-5.44] | 7.27 [7.81-6.73] | 6.75 [7.01-6.49] | 8.09 [8.59-7.59] | 8.73 [8.87-8.59] | 8.71 [8.77-8.65] |
| *Pseudomonas* spp. | 3.02 [3.37-2.66] | 3.93 [4.11-3.75] | 4.94 [5.48-4.40] | 5.16 [5.42-4.90] | - * | 5.55 [5.69-5.41] | 7.78 [7.84-7.72] |
| *L. gelidum* | 3.06 [3.41-2.71] | 4.87 [5.05-4.69] | 7.17 [7.71-6.63] | 7.42 [7.68-7.16] | 7.63 [8.13-7.13] | 8.21 [8.35-8.07] | 7.18 [7.24-7.12] |

Mean values with lower and upper confidence intervals. FW, food wrap packaging; MAP, modifed atmosphere packaging (CO_2_ 30% / O_2_ 70% ± 0.1 %); - ^*^ data under the detection limit.

**Supplementary R-commands 1.** Data fitting by primary and secondary models.

require(nlsMicrobio)

require(lattice)

require(deSolve)

require(growthrates)

baranyi

LOG10N ~ LOG10Nmax + log10((-1 + exp(mumax * lag) + exp(mumax *

t))/(exp(mumax * t) - 1 + exp(mumax * lag) * 10^(LOG10Nmax -

LOG10N0)))

environment: namespace:nlsMicrobio

data<-data.frame(t=c(0,24,48,72,96,120,168,216,288),LOG10N=c(1.49,1.55,1.94,2.45,3.83,6.37,9.16))

preview(formula=baranyi,data=data,start=list(lag=48,mumax=0.07,LOG10N0=1.49,LOG10Nmax=9.16))

primary<-nls(formula=baranyi,data=BT4data,start=list(lag=48,mumax=0.07,LOG10N0=1.49,LOG10Nmax=9.16))

sqrt<-as.formula("sqrtmumax~sqrt((T>Tmin)*muref*((T-Tmin)/(20-Tmin))^2)")

secondary<-data.frame(T=c(4,8,12),sqrtmumax=sqrt(c(0.07,0.10,0.24)))

preview(formula=sqrt,data=secondary,start=list(Tmin=-3.36,muref=0.53))

time<-c(0,48,96,120,216,288)

y<-grow_baranyi(time,c(y0=1.49,mumax=0.05,K=8.51,h0=6.24))

**Supplementary R-commands 2.** Analysis of covariance (ANCOVA) for bacterial growth parameters.

require(FactoMineR)

require(readr)

ancova<- read_delim("~/Desktop/Data.txt", "\t", escape_double = FALSE, trim_ws = TRUE)

test.ancova<-AovSum(counts~packaging*temperature+time+packaging:time+packaging:temperature+temperature:time,data=ancova)

test.ancova$Ftest

**Supplementary R-commands 3.** Modified Jameson-effect model without lag phase used for competition of two bacterial species.

library(nlsMicrobio)

t<-c(0,24,48,72,168,192,240,312)

data.specie1<-c(3.84,3.08,3.76,4.54,7.74,7.63,7.68,7.90)

rep.specie1<-rep(1,8)

specie1<-data.frame(t,rep.specie1,data.specie1)

data.specie2<-c(3.15,3.43,4.52,5.64,9.45,9.51,9.90,10.21)

rep.specie2<-rep(2,8)

specie2<-data.frame(t,rep.specie2,data.specie2)

names(specie1)<-c("t","flora","LOG10N")

names(specie2)<-c("t","flora","LOG10N")

rbind(specie1,specie2)

specie.1.vs.2<-rbind(specie1,specie2)

summary(specie.1.vs.2)

modified.jameson.baranyi.two.bacteria<-formula(LOG10N ~ (flora == 1) * ((t <= tmcp) * (LOG10N0_1 + mumax_1 * t/log(10) + log10(exp(-mumax_1 * t) * (1 - exp(-mumax_1 * lag_1)) + exp(-mumax_1 * lag_1))) + (t > tmcp) * (LOG10N0_1 + mumax_1 * tmcp /log(10) + log10(exp( mumax_1 * tmcp) * (1 - exp(-mumax_1 * lag_1)) + exp(-mumax_1 * lag_1)))) + (flora == 2) * ((t <= tmcp) * (LOG10N0_2 + mumax_2 * t/log(10) + log10(exp(-mumax_2 * t) * (1 - exp(-mumax_2 * lag_2)) + exp(-mumax_2 * lag_2))) + (t > tmcp) * (LOG10N0_2 + mumax_2 * tmcp /log(10) + log10(exp(-mumax_2 * tmcp) * (1 - exp(-mumax_2 * lag_2)) + exp(-mumax_2 * lag_2)))))

modified.jameson.buchanan.two.bacteria<-formula(LOG10N ~ (flora == 1) * ((t <= lag_1) * LOG10N0_1 + ((t > lag_1) & (t < tmcp)) * (LOG10N0_1 + mumax_1/log(10) * (t - lag_1)) + (t >= tmcp) * (LOG10N0_1 + mumax_1/log(10) * (tmcp - lag_1))) + (flora == 2) * ((t <= lag_2) * LOG10N0_2 + ((t > lag_2) & (t < tmcp)) * (LOG10N0_2 + mumax_2/log(10) * (t - lag_2)) + (t >= tmcp) * (LOG10N0_2 + mumax_2/log(10) * (tmcp - lag_2))))

modified.jameson.without.lag.two.bacteria<-formula(LOG10N ~ (flora == 1) * ((t < tmcp) * (LOG10N0_1 + mumax_1/log(10) * t) + (t >= tmcp) * (LOG10N0_1 + mumax_1/log(10) * tmcp)) + (flora == 2) * ((t < tmcp) * (LOG10N0_2 + mumax_2/log(10) * t) + (t >= tmcp) * (LOG10N0_2 + mumax_2/log(10) * tmcp)))

specie.1.vs.2.without.lag<-nls(modified.jameson.without.lag.two.bacteria, specie.1.vs.2,list(mumax_1=0.07,LOG10N0_1=3.84,tmcp=310,mumax_2=0.08,LOG10N0_2=3.15))

specie.1.vs.2.without.lag

summary(specie.1.vs.2.without.lag)

twocolors <- c("red","blue")

npoints <- 100

seq.t <- seq(0,max(specie.1.vs.2$t),length.out=npoints)

prednls3.1 <- predict(specie.1.vs.2.without.lag,data.frame(t=seq.t,flora=rep(1,npoints)))

prednls3.2 <- predict(specie.1.vs.2.without.lag,data.frame(t=seq.t,flora=rep(2,npoints)))

plot(specie.1.vs.2$t, specie.1.vs.2$LOG10N,col=twocolors[specie.1.vs.2$flora],xlab="t",ylab="LOG10N")

lines(seq.t,prednls3.1,col=twocolors[1])

lines(seq.t,prednls3.2,col=twocolors[2])

**Supplementary R-commands 4.** Modified Jameson-effect model without lag phase used for competition of three bacterial species.

library(nlsMicrobio)

t<-c(0,24,72,144,312)

A<-c(2.71,2.67,2.97,3.83,3.83)

fA<-rep(1,5)

metaA<-data.frame(t,fA,A)

B<-c(3.07,3.13,4.24,4.14,4.76)

fB<-rep(2,5)

metaB<-data.frame(t,fB,B)

C<-c(3.00,3.04,4.31,6.81,8.36)

fC<-rep(3,5)

metaC<-data.frame(t,fC,C)

names(metaA)<-c("t","flora","LOG10N")

names(metaB)<-c("t","flora","LOG10N")

names(metaC)<-c("t","flora","LOG10N")

rbind(metaA,metaB,metaC)

ABC<-rbind(metaA,metaB,metaC)

ABC

summary(ABC)

modified.jameson.without.lag.three.species<-formula(LOG10N ~ (flora == 1) * ((t < tmcp) * (LOG10N0_1 + mumax_1/log(10) * t) + (t >= tmcp) * (LOG10N0_1 + mumax_1/log(10) * tmcp)) + (flora == 2) * ((t < tmcp) * (LOG10N0_2 + mumax_2/log(10) * t) + (t >= tmcp) * (LOG10N0_2 + mumax_2/log(10) * tmcp)) + (flora == 3) * ((t < tmcp) * (LOG10N0_3 + mumax_3/log(10) * t) + (t >= tmcp) * (LOG10N0_3 + mumax_3/log(10) * tmcp)))

modified.jameson.without.lag.ABC<-nls(modified.jameson.without.lag.three.species,ABC,list(mumax_1=0.02,LOG10N0_1=2.71,tmcp=144,mumax_2=0.06,LOG10N0_2=3.07,mumax_3=0.01,LOG10N0_3=3.00)

overview(modified.jameson.without.lag.ABC)

predict(modified.jameson.without.lag.ABC)

threecolors <- c("red","blue","pink")

npoints <- 100

seq.t <- seq(0,max(ABC$t),length.out=npoints)

prednls3.1 <-predict(modified.jameson.without.lag.ABC,data.frame(t=seq.t,flora=rep(1,npoints)))

prednls3.2 <- predict(modified.jameson.without.lag.ABC,data.frame(t=seq.t,flora=rep(2,npoints)))

prednls3.3 <- predict(modified.jameson.without.lag.ABC,data.frame(t=seq.t,flora=rep(3,npoints)))

plot(ABC$t,ABC$LOG10N,col=threecolors[ABC$flora],xlab="t",ylab="LOG10N")

lines(seq.t,prednls3.1,col=threecolors[1])

lines(seq.t,prednls3.2,col=threecolors[2])

lines(seq.t,prednls3.3,col=threecolors[3])

**Supplementary R-commands 5.** Lotka-Volterra model used for competition of three bacterial species.

library(deSolve)

library(nlsMicrobio)

library(car)

LVmodel.three<-function(Time,State,Pars){

with(as.list(c(State,Pars)),{

dx=mumax1*(1-((x+alpha*delta*3)/Nmax1))

dy=mumax2*(1-((y+beta*epsilon*3)/Nmax2))

dz=mumax2*(1-((z+gamma*zeta*3)/Nmax3))

return(list(c(dx,dy,dz)))

})

}

Pars<-c(mumax1=0.13,mumax2=0.32,mumax3=0.15,alpha=0.3833,beta=-0.3382,gamma=0.2456,delta=-0.2456,epsilon=0.1546,zeta=-0.1345,Nmax1=8.83,Nmax2=8.87,Nmax3=6.78)

State<-c(x=3.84,y=4.00,z=3.85)

Time<-seq(0,13,by=1)

three.bacteria.LVmod<-as.data.frame(ode(func=LVmodel.three,y=State,parms=Pars,time=Time))

three.bacteria.LVmod

summary(three.bacteria.LVmod)

matplot(three.bacteria.LVmod[,-1],type="l",xlab="time",ylab="population")
